# Genetic Architecture of Acute Myocarditis and the Overlap With Inherited Cardiomyopathy

**DOI:** 10.1161/CIRCULATIONAHA.121.058457

**Published:** 2022-09-26

**Authors:** Amrit S. Lota, Mark R. Hazebroek, Pantazis Theotokis, Rebecca Wassall, Sara Salmi, Brian P. Halliday, Upasana Tayal, Job Verdonschot, Devendra Meena, Ruth Owen, Antonio de Marvao, Alma Iacob, Momina Yazdani, Daniel J. Hammersley, Richard E. Jones, Riccardo Wage, Rachel Buchan, Fredrik Vivian, Yakeen Hafouda, Michela Noseda, John Gregson, Tarun Mittal, Joyce Wong, Jan Lukas Robertus, A. John Baksi, Vassilios Vassiliou, Ioanna Tzoulaki, Antonis Pantazis, John G.F. Cleland, Paul J.R. Barton, Stuart A. Cook, Dudley J. Pennell, Pablo Garcia-Pavia, Leslie T. Cooper, Stephane Heymans, James S. Ware, Sanjay K. Prasad

**Affiliations:** National Heart & Lung Institute (A.S.L., P.T., R.W., S.S., B.P.H., U.T., A.d.M., A.I., M.Y., M.J.H., R.E.J., R.W., R.B., M.N., J.L.R., A.P., J.G.F.C., P.J.R.B., D.J.P., J.S.W., S.K.P.), Imperial College London, UK.; Epidemiology and Biostatistics, School of Public Health (D.M., I.T.), Imperial College London, UK.; MRC London Institute of Medical Sciences (P.J.R.B., S.A.C., J.S.W.), Imperial College London, UK.; Royal Brompton & Harefield Hospitals, Guy’s and St. Thomas’ NHS Foundation Trust, London, UK (A.S.L., P.T., R.W., S.S., B.P.H., U.T., A.d.M., A.I., M.Y., M.J.H., R.E.J., R.W., R.B., F.V., Y.H., T.M., J.W., J.L.R., A.J.B., A.P., P.J.R.B., D.J.P., J.S.W., S.K.P.).; Centre for Heart Failure Research, Cardiovascular Research Institute Maastricht, Maastricht University Medical Centre, the Netherlands (M.R.H., J.V., S.H.).; London School of Hygiene and Tropical Medicine, UK (R.O., J.G.).; Norfolk and Norwich University Hospital and University of East Anglia, Norwich, UK (V.V.).; Robertson Centre for Biostatistics, University of Glasgow, UK (J.G.F.C.).; National Heart Centre Singapore and Duke-National University of Singapore (S.A.C.).; Heart Failure and Inherited Cardiac Diseases Unit, Department of Cardiology, Hospital Universitario Puerta de Hierro, CIBERCV, Madrid, Spain (P.G.-P.).; Universidad Francisco de Vitoria, Pozuelo de Alarcon, Spain (P.G.-P.).; Centro Nacional de Investigaciones Cardiovasculares, Madrid, Spain (P.G.-P.).; Department of Cardiovascular Medicine, Mayo Clinic, Jacksonville, FL (L.T.C.).

**Keywords:** arrhythmogenic right ventricular dysplasia, cardiomyopathy, dilated, connectin, death, sudden, cardiac, desmoplakins, heart failure, myocarditis

## Abstract

Acute myocarditis is an inflammatory condition that may herald the onset of dilated cardiomyopathy (DCM) or arrhythmogenic cardiomyopathy (ACM). We investigated the frequency and clinical consequences of DCM and ACM genetic variants in a population-based cohort of patients with acute myocarditis.

**Methods:**

This was a population-based cohort of 336 consecutive patients with acute myocarditis enrolled in London and Maastricht. All participants underwent targeted DNA sequencing for well-characterized cardiomyopathy-associated genes with comparison to healthy controls (n=1053) sequenced on the same platform. Case ascertainment in England was assessed against national hospital admission data. The primary outcome was all-cause mortality.

**Results:**

Variants that would be considered pathogenic if found in a patient with DCM or ACM were identified in 8% of myocarditis cases compared with <1% of healthy controls (*P*=0.0097). In the London cohort (n=230; median age, 33 years; 84% men), patients were representative of national myocarditis admissions (median age, 32 years; 71% men; 66% case ascertainment), and there was enrichment of rare truncating variants (tv) in ACM-associated genes (3.1% of cases versus 0.4% of controls; odds ratio, 8.2; *P*=0.001). This was driven predominantly by *DSP*-tv in patients with normal LV ejection fraction and ventricular arrhythmia. In Maastricht (n=106; median age, 54 years; 61% men), there was enrichment of rare truncating variants in DCM-associated genes, particularly *TTN*-tv, found in 7% (all with left ventricular ejection fraction <50%) compared with 1% in controls (odds ratio, 3.6; *P*=0.0116). Across both cohorts over a median of 5.0 years (interquartile range, 3.9–7.8 years), all-cause mortality was 5.4%. Two-thirds of deaths were cardiovascular, attributable to worsening heart failure (92%) or sudden cardiac death (8%). The 5-year mortality risk was 3.3% in genotype-negative patients versus 11.1% for genotype-positive patients (*P*_adjusted_=0.08).

**Conclusions:**

We identified DCM- or ACM-associated genetic variants in 8% of patients with acute myocarditis. This was dominated by the identification of *DSP*-tv in those with normal left ventricular ejection fraction and *TTN*-tv in those with reduced left ventricular ejection fraction. Despite differences between cohorts, these variants have clinical implications for treatment, risk stratification, and family screening. Genetic counseling and testing should be considered in patients with acute myocarditis to help reassure the majority while improving the management of those with an underlying genetic variant.

**Clinical Perspective**

**What Is New?**

- In this population-based study (n=336), ≈1 in 13 patients (8%) with acute myocarditis was found to have a rare variant in a gene robustly linked to dilated cardiomyopathy or arrhythmogenic cardiomyopathy that would be reported as likely pathogenic in a patient with cardiomyopathy compared with <1% of healthy controls (n=1053).
- This finding was dominated by truncating variants in titin in patients with reduced left ventricular ejection fraction and desmoplakin in those with preserved left ventricular ejection fraction at presentation.
- Over a median follow-up of 5 years, there was a trend toward greater all-cause mortality in genotype-positive patients compared with genotype-negative patients (*P*=0.08).

**What Are the Clinical Implications?**

- This study highlights the potential role of genetic sequencing in patients presenting with acute myocarditis and supports the concept that genotype-positive individuals may remain phenotypically silent until the occurrence of an environmental trigger.
- These findings may help to explain some of the considerable heterogeneity in clinical outcomes in acute myocarditis, with some individuals requiring cardiac transplantation or device implantation but others recovering fully with no long-term sequelae.
- Incorporation of routine genetic testing may inform risk stratification and clinical management, including the need for ongoing surveillance and family screening when cardiomyopathy-associated genetic variants are present.

Acute myocarditis affects ≈20 per 100 000 people each year and is often thought to be triggered by a viral infection.^1^ Myocarditis affects people of all ages. Clinical presentation is heterogeneous, and markers of adverse risk are poorly defined. Although spontaneous recovery occurs in two-thirds of patients, left ventricular (LV) dysfunction persists in the remainder, and postmortem studies implicate myocarditis in 3% to 12% of all sudden cardiac deaths (SCDs).^2,3^ There are no previous genetic studies in unselected adult patients with acute myocarditis. However, familial cases of recurrent myocarditis, selected case series, pediatric studies, and a recent retrospective cohort study all suggest the possibility of an underlying genetic susceptibility linked to inherited cardiomyopathy, which may determine the clinical course.^4–12^ These reports have sparked a new interest into a possible underlying genetic basis of myocarditis, as outlined in a scientific statement earlier this year.^13^

We report the first large-scale international genetic evaluation of patients with acute myocarditis to determine the frequency of genetic variants associated with inherited cardiomyopathies and, in particular, if truncating variants (tvs) in *TTN*, a sarcomeric protein, are more common in those who develop LV dysfunction. We hypothesized that myocarditis may act as an environmental modifier, triggering phenotypic expression of dilated cardiomyopathy (DCM) in previously healthy but genotype-positive individuals attributable to an abnormal response to hemodynamic stress, as reported in other clinical settings.^14–16^ Phenotypic overlap between arrhythmogenic cardiomyopathy (ACM) and myocarditis has also been observed in small single-center studies with much uncertainty about this complex relationship.^17,18^ Histological features of myocarditis found in patients with ACM suggest that an infective or inflammatory mechanism could represent a second “hit” driving the onset and progression of ACM or may simply be a “hot phase” in its natural history.^19–21^ More recent evidence also suggests a vulnerability to acute myocarditis determined by the presence of underlying genetic variants in cardiomyopathy-associated proteins.^22,23^

There is a clear unmet need to improve our scientific understanding of the genetic architecture of acute myocarditis and potential overlaps with different forms of inherited cardiomyopathy, particularly given the increasing burden of myocarditis, to improve clinical management.^24^

## Methods

### Cohorts

Four cohorts were included in this study.

**Figure 1.**
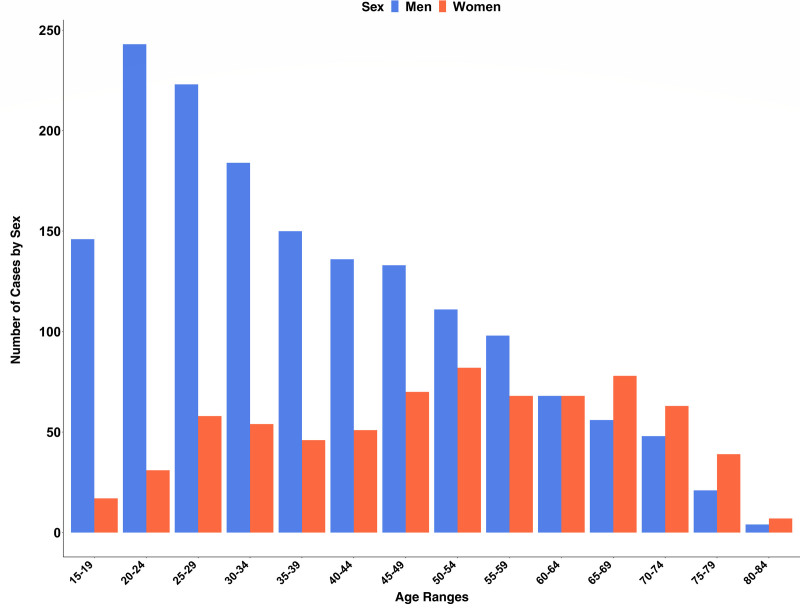
**Bar chart showing the age and sex distributions of patients with a primary or secondary diagnosis of acute myocarditis Across all hospitals in National Health Service England from 2016 to 2018.** Age indicated in years.

In cohort 1, 230 cases with myocarditis were recruited from hospitals across Northwest London (Figure 1A) and evaluated by cardiovascular magnetic resonance (CMR) or immunohistopathology of myocardial tissue by European Society of Cardiology criteria.^25^ Of these, 114 were consecutively recruited <14 days after acute hospitalization, and 116 were retrospectively identified with CMR or biopsy-confirmed acute myocarditis. Exclusion criteria were coronary artery disease (>50% luminal stenosis) and congenital heart disease.

Cohort 2 consisted of 1053 community based healthy volunteers in London with no history of medical illness and no regular medication who were recruited through advertisements in the national media. None were known to have had previous myocarditis. Normal cardiac structure and function were confirmed on noncontrast CMR.

Cohort 3 included 106 myocarditis cases in the Netherlands confirmed on endomyocardial biopsy (EMB) within 6 months of acute presentation with suspected myocarditis by European Society of Cardiology criteria.^25^ CMR was also performed in 103 cases. Exclusion criteria matched those in cohort 1.

Cohort 4 comprised 141 456 unrelated individuals from an international reference population Genome Aggregation Database (gnomAD).^26^

All participants in cohorts 1 through 3 provided written informed consent as approved by the National Research Ethics Service (references 09/H0504/104+5 and 09/H0707/69) or Medical Ethical Committee of Maastricht University. The study was performed according to the Declaration of Helsinki.

### National Hospital Admission Data in England

To assess whether the London myocarditis cohort was representative of national-level myocarditis admissions, we obtained patient-level data from the Hospital Episodes Statistics database curated by National Health Service (NHS) Digital. This included all patients >16 years of age admitted to hospital in England with a primary or secondary diagnosis of myocarditis, defined by World Health Organization’s *International Classification of Diseases, 10th Revision* code I40, I41, or I51.4, from June 17, 2016, to June 18, 2018. Finished consultant episodes were aggregated to represent hospital admissions. Baseline patient characteristics were evaluated both on a national level and within the pool of study recruitment sites across London identified by their individual NHS hospital provider codes. Case ascertainment was then measured as the ratio of the number of cases recruited to the number admitted with myocarditis in the region served by these hospitals (comprising 2.3 million people) during the study time span from 2016 to 2018. Global Burden of Disease 2019 subnational data for all 33 London boroughs were also extracted to assess regional differences in myocarditis prevalence (Supplemental Material).^27^

### Baseline Evaluation of UK Cohorts

Participants were recruited during a single study visit for blood collection, ECG, and CMR. Medical history and family pedigrees were obtained with structured proformas. SCD was defined as unexpected death within 1 hour of cardiac symptoms in the absence of progressive cardiac deterioration, during sleep, or within 24 hours of last being seen alive. Blood samples were processed with Biobank procedures (Supplemental Appendix). CMR was performed with standardized protocols including assessment of Lake Louise criteria (Supplemental Appendix). An independent expert reviewed a random subset of 30 CMR studies to confirm that the clinical diagnosis of acute myocarditis was accurate. All CMR analyses were performed blinded to genotype, which was not available until a later date. EMB was performed in a subset of cases when clinically indicated.

### Baseline Evaluation of the Netherland Cohorts

Participants were enrolled after routine clinical evaluation with medical history, ECG, venous blood collection, and echocardiography. EMB was performed with a standardized protocol, including assessment of immunohistochemistry.^25^ Contrast-enhanced CMR was performed in a subset of cases.

### DNA Extraction and Sequencing

DNA extraction was performed in both centers from whole blood with the use of automated platforms followed by targeted sequencing on Illumina NextSeq platforms (Supplemental Material). Variants were annotated and filtered according to a customized bioinformatics pipeline.

### Follow-Up CMR Evaluation

Patients recruited prospectively (n=114) in London underwent serial follow-up with CMR at 3 and 12 months to understand the impact of identified variants on cardiac function.

### Sample Size Calculation

Given a 1% background prevalence of rare *TTN*-tv in healthy populations, 70 patients per group (myocarditis with/without LV dysfunction) would yield 90% power to detect a significant enrichment in the proportion with *TTN*-tv if observed in ≈15% of cases, as found in DCM and peripartum cardiomyopathy, at the 5% significance level. We aimed to recruit 210 patients, with one-third expected to progress to DCM. A recent study of pediatric and adult patients with myocarditis identified deleterious cardiomyopathy-associated variants in ≈16% of patients, suggesting that <60 patients per group may be required.^22^

### Statistical Analysis

Baseline characteristics were compared with the Mann-Whitney *U* test or Fisher exact test. Although the Illumina cardiac panel captured many genes linked to DCM and ACM, we performed a prespecified, focused analysis on protein-altering variants in 11 DCM (*BAG3, DES, LMNA, MYH7, PLN, RMB20, SNC5A, TNNC1, TNNT2, TPM1*, and *TTN*) and 5 ACM (*DSC2, DSG2, DSP, PKP2*, and *JUP*) genes definitively associated with disease and restricted analyses to variant classes known to cause disease and consistent with known mechanisms of pathogenesis (Table S1).^28,29^ Truncating variants included nonsense, frameshift, or essential splice-site variants. Rare variants were defined with overall minor allele frequency <0.0001 and the highest filtering allele frequency in gnomAD (popmax95).

We first compared the prevalence of rare variants by disease in cases and controls matched technically (cohorts 1 and 2) and, when possible, by ethnicity (self-reported White, confirmed by principal component analysis; Figure S8) with sensitivity analyses to determine the effect size if all ethnicities were included. We prespecified 3 association tests: between myocarditis and rare variants in DCM-associated genes, ACM-associated genes, and *TTN*-tv. We analyzed truncating and nontruncating variants separately because of known differences in background signal. We then performed secondary analyses of individual genes. The Bonferroni correction was used to reduce the chances of obtaining false-positive results (type I errors). Pathogenicity was assigned with the use of American College of Medical Genetics and Genomics criteria as the final step.^30^ We report the initial output of the CardioClassifier semiautomated decision support tool to identify variants that would be reported as pathogenic or likely pathogenic if identified in an individual with DCM or ACM (without additional manual curation).^31^ Statistical analyses were conducted with the R software environment (version 4.0.3) with the tidyverse and rstatix packages.

An extended list of genes linked to DCM and ACM with varying levels of evidence was also explored, including *DMD* as a phenocopy of myocarditis^32^ and motivated by animal studies demonstrating that dystrophin-deficient mice were more susceptible to virus-mediated cardiomyopathy.^33^

### Clinical Follow-Up and Outcomes

Major cardiovascular outcomes were assessed by genetic status across both cohorts. The prespecified primary outcome was all-cause mortality (cause of death was established from death certificates and postmortem examinations). Secondary composite outcomes consisted of major arrhythmia (ventricular tachycardia, aborted SCD, implantable cardioverter defibrillator, or second/third-degree heart block) and major heart failure events (heart transplantation, LV assist device implantation, or heart failure hospitalization). Follow-up duration was calculated from the baseline CMR or EMB date and censored at the first event or last patient contact. All clinical data were adjudicated by an independent committee of cardiologists.

Cumulative incidence curves were generated for outcomes with event times measured from the baseline date. Associations between the genetic status and outcomes were analyzed with multivariable Cox proportional hazard modeling adjusted for known important predictors of outcome, specifically age and sex. Results are presented as hazard ratios with 95% CIs. Statistical analyses were performed with Stata version 14 (StataCorp). A value of *P*<0.05 was taken as significant.

### Data Sharing

Anonymized participant data and detailed genetic findings are available from the time of publication. Appropriate institutional data transfer agreements will be required. Requests should be made through email to the corresponding author, along with an analysis proposal.

## Results

### Cohort Review

In London, the median age at presentation was 33 years (interquartile range [IQR], 25–45 years), 84% were men, 85% reported a recent viral illness, and 95% were New York Heart Association class I/II; median LV ejection fraction (LVEF) by CMR was 63% (IQR, 57%–67%; Table 1, Figure 1, and Table S3). All patients had edema and LGE present, consistent with Lake-Louise criteria for acute myocarditis, and 12 cases underwent EMB (4%). A family history of SCD in a first-degree relative was reported in 2%, and myocarditis was reported in 3%. In the control cohort, the median age was 35 years (IQR, 27–48 years), and 44% were men. A family history of SCD, but not myocarditis, was reported in 1%. In the Maastricht cohort, the median age was 54 years (IQR, 44–54 years), 61% were men, and 62% were in New York Heart Association class I/II; median LVEF was 30% (IQR, 21%–40%) by echocardiography and 36% (IQR, 24%–45%) by CMR (in 103 cases). A family history of confirmed DCM or SCD was reported in 2%. There were no significant differences in family history between genotype-positive and genotype-negative cases in either London or Maastricht.

**Table 1.**
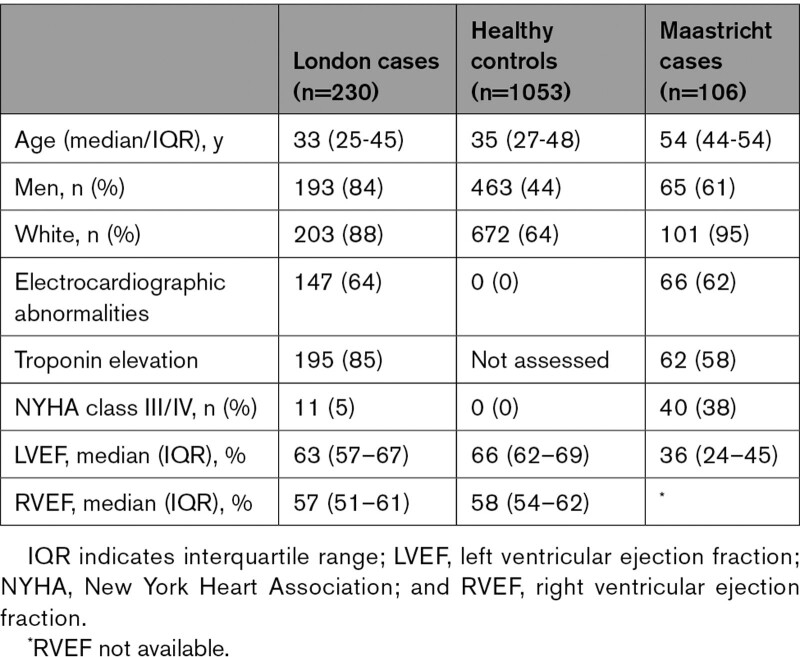
Baseline Demographics of the London and Maastricht Acute Myocarditis Cohorts at First Clinical Presentation Compared With Healthy Controls

### National Admission Data

**Figure 2.**
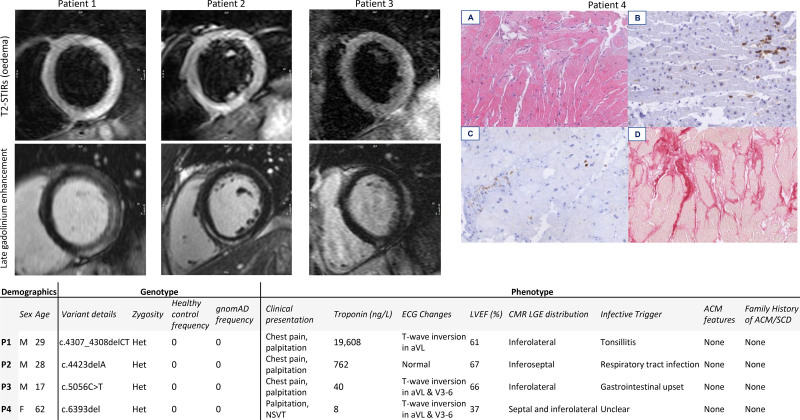
**Detailed review of the genotype and phenotype of all cases with truncating variants of known pathogenicity in DSP.** Cardiac magnetic resonance short-axis images (T2-STIR sequence for myocardial oedema, **top**; late gadolinium enhancement, **bottom**) are presented for the 3 cases from London and histopathology for the 1 case from Maastricht. Images are taken at 100 µm and demonstrate staining for (**A**) hematoxylin-eosin, (**B**) CD45, (**C**) CD68, and (**D**) Sirius red. ACM indicates arrhythmogenic cardiomyopathy; FHx, family history; gnomAD, Genome Aggregation Database; Het, heterogeneous; LVEF, left ventricular ejection fraction; P, patient; SCD, sudden cardiac death; and Trop, troponin.

In total, there were 2353 national admissions attributable to acute myocarditis over the 2-year study period across NHS England (69% men; median age, 40 years; IQR, 27–55 years). Men were significantly younger than women on admission to hospital (median age, 35 years [IQR, 25–50 years] versus 52 years [IQR, 38–65 years]; *z* score, −9.633; *P*<0.001; Figure 2). Within the recruitment network, there were 175 admissions with a primary diagnosis of acute myocarditis (71% men; median age, 32 years; IQR, 23–52 years). Therefore, case ascertainment was calculated as 66% for the prospectively recruited patients in the London cohort.

### Disease-Based Burden

In London, 11 patients (4.8%) had rare truncating variants in prespecified cardiomyopathy-associated genes (Table 2; listed in Table S7). There was a significant excess of truncating variants in ACM genes among cases compared with controls (3.0% versus 0.4%; odds ratio [OR], 8.2 [95% CI, 2.4–28.4]; *P*<0.001; Figure S9) but no significant enrichment in DCM genes in aggregate or of *TTN*-tv. There were no significant differences in genetic findings between patients recruited prospectively and those recruited retrospectively (Figure S10).

**Table 2.**
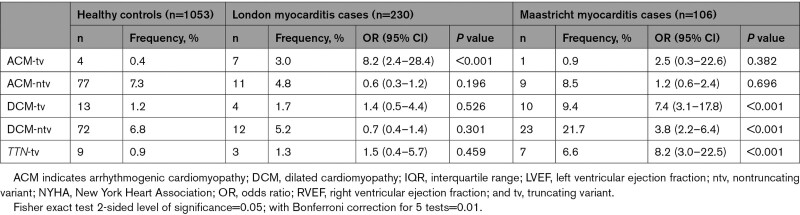
Odds Ratios and Fisher Exact Test Results for Significance of the Excess of Rare Genetic Variation in Cases With Acute Myocarditis in London and Maastricht Versus Healthy Controls in Key DCM- and ACM-Associated Genes, Including *TTN*-tv Specifically as per Our A Priori Hypothesis

In Maastricht, 11 patients (10.4%) had rare truncating variants in the prespecified genes (listed in Table S8). In contrast to London, there was a significant excess of truncating variants in DCM genes compared with controls (9.4% versus 1.2%; OR, 7.4 [95% CI, 3.1–17.8]; *P*<0.001), *TTN*-tv specifically (6.6% versus 0.9%; OR, 8.2 [95% CI, 3.0–22.5]; *P*<0.001), but no significant enrichment in ACM genes.

### Gene-Based Burden

In London, the gene with the highest prevalence of truncating variants was *DSP*. There were 3 *DSP*-tvs in 3 unrelated myocarditis cases (1.3% prevalence) and none in 1053 healthy controls (*P*_Fisher_=0.006; ∆+1.3%; Table S4). No other individual ACM gene demonstrated a statistically significant association, but the signal was not solely attributable to *DSP* because truncating variants in the other 4 ACM genes were also enriched in myocarditis cases in aggregate (*P*_Fisher_=0.039; ∆+1.4%). Three patients had *TTN*-tv with reduced LVEF (median, 36%). We found no significant enrichment in the prespecified DCM genes in cases compared with controls, either in aggregate or individually (Table S5).

In addition to our highly phenotyped controls, we compared the burden of variants between cases and gnomAD, which provided a larger population for increased power albeit with increased technical heterogeneity. As before, there was an excess of *DSP*-tv in cases compared with gnomAD (*P*_Fisher_=0.001; ∆+1.2%; Table S6).

In Maastricht, the gene with the highest prevalence of truncating variants was *TTN.* There were 7 cases with *TTN*-tv (6.6% prevalence), all of whom exhibited LV dysfunction, compared with 0.9% in our controls (OR, 8.2 [95% CI, 3.0–22.5]; *P*<0.001). One case with no family history had a likely pathogenic *DSP*-tv, presented with nonsustained ventricular tachycardia, underwent EMB (Figure S11), and subsequently received an implantable cardioverter defibrillator.

### Variant Pathogenicity

In London, 10 cases (4.3%) carried variants that would be considered likely pathogenic if identified in an individual with cardiomyopathy, of which 7 were in ACM-associated genes, consistent with earlier findings (Figure S12). A detailed retrospective review of clinical data was performed confirming normal right ventricular structure and function and negative family history for these individuals (Figure 2 and Figures S13–S16). In Maastricht, 17 cases (16%) carried variants that would be considered pathogenic if identified in an individual with cardiomyopathy, all of which were in DCM-associated genes apart from the single case with a *DSP*-tv.

### Extended Gene Panel

Exploratory analyses were performed on nondesmosomal genes reported to be associated with ACM (*TMEM43, DES, JPH2, TGFB3*, and *CTTNA3*) and *DMD*. A rare frameshift variant of uncertain significance was identified in desmin in 1 case, which was absent from both control cohorts. Five individuals with no skeletal myopathy had missense *DMD* variants of uncertain significance but potentially deleterious to dystrophin stability.

### Serial Change in LV Structure and Function

No significant changes in median LVEF or LV end-diastolic volume at 3 or 12 months were observed for prospective cases in London in the presence or absence of a variant (Figure S17).

### Combined Genetic Findings

**Figure 3.**
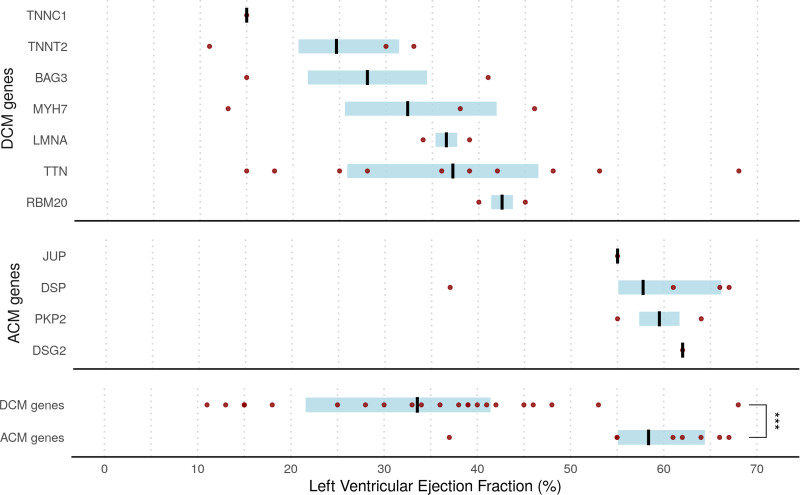
**Distribution of LVEF assessed by cardiac magnetic resonance in patients with acute myocarditis recruited in London (cohort 1; n=230) and Maastricht (cohort 4; n=106) stratified by presence of likely pathogenic variants in ACM- and DCM-associated genes.** Black lines indicate median; blue shading shows interquartile range. Dots refer to individual patients. Note that genes with a single patient affected have left ventricular ejection fraction (LVEF) shown as an absolute value (applies to *DSG2, JUP*, and *TNNC1*). ACM indicates arrhythmogenic cardiomyopathy; and DCM, dilated cardiomyopathy.

Overall, 22.9% of cases (77 of 336) carried rare protein-altering variants in cardiomyopathy-associated genes compared with a prevalence of 11% in gnomAD (*P*<0.0001). There was enrichment of rare truncating variants in both DCM and ACM genes (4.2% and 2.4% of cases, respectively). In total, 8% of cases (27 of 336) carried likely pathogenic variants in DCM and ACM genes. DCM variants were seen predominantly in patients with LV dysfunction (median LVEF, 39% versus 60%; *P*<0.0001). Patients with variants in DCM-associated genes were also older (median age, 52 years versus 38 years; *P*=0.0014). In contrast, ACM variants were seen in patients with preserved LV function. LVEF assessed by CMR could therefore be stratified by genotype in those with a variant (Figure 3). Recognizing differences in cohorts, we additionally matched patients for New York Heart Association class and confirmed that all patients with ACM variants were in class I/II. In total, *TTN*-tvs were present in 10 of 336 cases (3%), representing enrichment compared with our healthy controls (0.9%; OR, 3.6 [95% CI, 1.4–8.8]; *P*=0.0116).

### Clinical Outcomes

**Figure 4.**
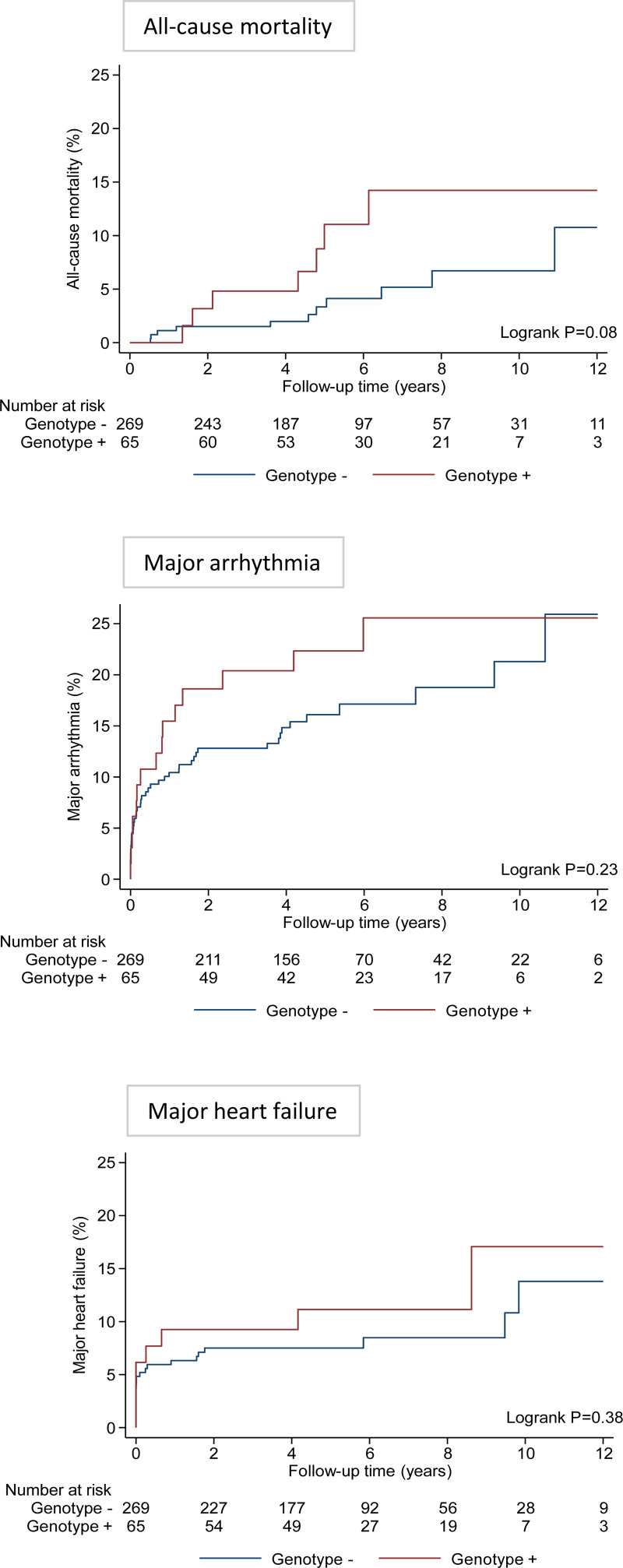
**Cumulative incidence curves for the study end points over follow-up by the presence or absence of a protein-altering variant across both cohorts.** Major arrhythmia composite includes hemodynamically unstable ventricular tachycardia, aborted sudden cardiac death, implantable cardioverter defibrillator, or heart block (second or third degree). Major heart failure composite includes heart transplantation, left ventricular assist device implantation, or heart failure hospitalization.

Over a median follow-up of 5.0 years (IQR, 3.9–7.8 years), there were 18 (5.4%) deaths, of which 12 were cardiovascular. The causes of cardiovascular death were heart failure (11 patients, 92%) and SCD (1 patient, 8%). In total, 3 patients underwent orthotopic cardiac transplantation, of whom 1 died (*BAG3* variant identified). The 5- and 10-year risks of all-cause mortality were 3.3% and 6.7% for genotype-negative patients compared with 11.1% and 14.2% for genotype-positive patients (*P*=0.08 after adjustment for age at presentation and sex; Figure 4).

The outcome for patients with or without an ACM gene variant was similar, although compared with controls there was a trend toward a greater burden of life-threatening arrhythmia in those with an ACM variant (∆+8%; *P*=0.21; Table S9). For patients with reduced LV function, all-cause mortality was higher for those who had a DCM genetic variant (1 with *BAG3*, 1 with *LMNA*, 2 with *MYH7*, 2 with *TTN*) compared those with no variant (17.6% versus 4.2%; OR, 5.0 [95% CI, 1.8–13.8]; *P*=0.004).

In the Maastricht cohort (median follow-up duration, 7.4 years; IQR, 5.5–9.6 years), the risk of major arrhythmia or death was higher in patients who had a DCM gene variant compared with those who did not (OR, 4.0 [95% CI, 1.3–12.4]; *P*=0.02; and OR, 2.5 [95% CI, 1.1–5.3]; *P*=0.04, respectively; Table 3). In the London cohort (median follow-up, 4.5 years; IQR, 3.5–6.2 years), there were no significant associations between genotype status and clinical events.

**Table 3.**
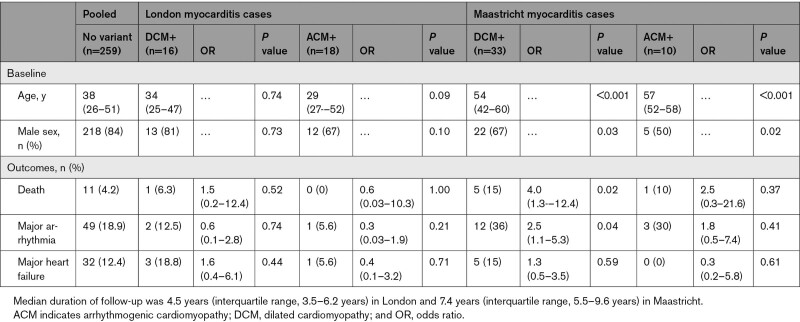
Clinical Characteristics and Outcomes of Cases With Acute Myocarditis With and Without Protein-Altering Variants in DCM- or ACM-Associated Genes (Truncating and Nontruncating) in Both Cohorts Compared With the Pooled Cohort of Patients With No Variants (ORs and Fisher Exact Test Results)

## Discussion

Genetic evaluation identified pathogenic or likely pathogenic variants in cardiomyopathy-associated genes in 8% of patients presenting with acute myocarditis, demonstrating an overlap between myocarditis and inherited cardiomyopathy. The Northwest London cohort captured two-thirds of all adult admissions recorded in NHS records within this population of 2.3 million, thereby approximating the base of the pyramid of disease and making it the first substantial population-representative study of its kind for myocarditis. These patients were typically young, presenting with chest pain, troponin elevation, and a normal LVEF. In this population, we identified ACM-tvs in 3.0% of cases (compared with 0.4% of controls), of which *DSP*-tvs were the most common. In contrast, the Maastricht cohort was older and more often had reduced LVEF and symptoms of heart failure. In this population, DCM variants were identified in 9.4% of cases (compared with 1.2% of controls), most commonly *TTN*-tv, with a trend toward higher mortality.

### Myocarditis and ACM

ACM as a clinical entity is recognized to have subclinical (concealed) phenotypes, hot phases of disease activity, and isolated LV involvement.^34,35^ Families affected by myocarditis show enrichment of *DSP*-tv, suggesting that myocarditis may be the first clinical expression of an underlying ACM.^6,7^ Similarly, a case series of 16 unrelated patients with myocarditis with right ventricular abnormalities or ventricular arrhythmia found ACM variants in 56%.^8^ Seen from another perspective, 15% of patients with cardiomyopathy with known *DSP* variants experienced episodes of myocardial injury akin to myocarditis, suggesting a novel entity known as desmoplakin cardiomyopathy, characterized by preserved ventricular function and subepicardial fibrosis.^36^ The largest existing genetic study of 117 pediatric and adult cases of biopsy-confirmed myocarditis reported *DSP* variants as the second most common finding in 3 cases (2.6%); *TTN*-tvs were present in 7 cases (6%).^22^ Most recently, a retrospective cohort study of 28 patients with myocarditis identified pathogenic or likely pathogenic variants linked to cardiomyopathy in 18% of patients.^12^ Of those with severe myocarditis, defined by LVEF <30% (n=12), variants were identified in 33% (n=4, *FLNC, RBM20, BAG3, BAG3*), whereas in those with nonsevere myocarditis (n=16), 1 patient (6%) had a pathogenic variant in *DSP* with a family history of cardiomyopathy.

In our London cohort, likely pathogenic desmosomal variants occurred in 3% with typical clinical and imaging features of myocarditis (but not of ACM), and none reported a family history of premature cardiac disease or cardiomyopathy. Previous studies have reported a retrospective diagnosis of ACM ≈1.8 years (IQR, 2.7 years) after initial presentation with myocarditis; therefore, in the absence of genetic testing, extended follow-up is required.^37^ Although the genetic yield of ACM was low, thereby resulting in an increased level of uncertainty about our effect estimates, our study represents the largest cohort of patients with myocarditis to undergo genetic evaluation to date. This finding was highly significant and exceeds previous clinical studies and postmortem genetic evaluation. Identification of such variants could substantially alter management and clinical outcomes of patients and their families.

Additional work should determine whether the underlying ACM genetic defect renders the heart more susceptible to viral infection or if infection itself drives further adverse remodeling. Postmortem studies of patients with ACM have revealed T-cell infiltrates and other features of myocarditis,^19,20^ including presence of cardiotropic viruses.^38^ Mechanistically, cytokine exposure can disrupt desmosomal plakoglobin,^39^ and enteroviral protease-2A can cleave dystrophin, leading to functional impairment.^40^ Autoimmunity may also play a role, with anti-heart and anti–intercalated disk antibodies present in sporadic cases with ACM and relatives.^25,41^

### Myocarditis and DCM

We identified *TTN*-tvs in 6.6% of patients in Maastricht and 1.3% in London (compared with 0.9% in controls), with a higher prevalence in patients with reduced LVEF. This is the largest prospective study to date and supports our original hypothesis of a shared genetic predisposition with familial DCM attributable to enrichment of *TTN*-tvs.^14–16^ In myocarditis, edema, fibrosis, endothelial dysfunction, and altered electric conduction, along with activation of inflammatory cascades and immune responses, may conspire to impair LV function in predisposed individuals. In animal models, increases in interleukin-6 were associated with reduced phosphorylation of titin and cardiac dysfunction, preventable by interleukin-6 receptor blockade.^42,43^

Variations in observed DCM gene prevalence between the London and Maastricht cohorts likely reflect differences in clinical presentation, although geographic differences may have influenced viral etiology and clinical course. In London, most patients with myocarditis had pseudoinfarct presentations, whereas the Maastricht cohort was accrued mainly after myocardial biopsy findings, thereby potentially biasing the sample toward those with a reduced LVEF. Our findings were consistent with the 6% *TTN*-tv prevalence reported in a smaller cohort that was also ascertained on the basis of immunohistological criteria.^22^ Of note, among patients who had LVEF assessed during their acute illness, 66% had an LVEF <40%; potentially deleterious variants were found in 12 of 55 cases (21%) compared with 2 of 28 (7%) with LVEF >40%. In summary, differences in the method of case ascertainment may account for the observed differences in gene prevalence. We believe that in most countries few patients with myocarditis, especially those who do not have a reduced LVEF, have a myocardial biopsy taken. Although there are controversy and equipoise on the use of myocardial biopsy, we believe this highlights the strength of our population-representative London cohort, which was not subject to enrichment of those with more severe phenotypes, typical of studies in tertiary centers.

We found that patients with DCM variants tended to have a worse outcome with greater mortality after adjustment for age and sex compared with those with no variants or ACM variants. This novel finding was not uniquely driven by *TTN*-tv.^44^ Although there were no significant differences in clinical outcomes by genotype status, our study was not powered to assess clinical outcomes, and the shorter follow-up in London may have contributed to the lower event rate in patients with ACM variants. Nevertheless, our findings are consistent with a previous pediatric study in which pathogenic or likely pathogenic variants were identified in 9 of 42 cases (22%), were more common in those with reduced LVEF, and were associated with lower event-free survival.^11^ This was also the case in a recent retrospective study in which 33% of patients with myocarditis with LVEF <30% harbored a genetic variant compared with 6% of those with LVEF >30%.^12^ Therefore, knowledge of the presence or absence of such variants may play a key role in prognosis and risk stratification. These interesting trends should be explored further in multicenter studies over greater follow-up durations.

We recognize several limitations in this study. First, we did not include people who died suddenly as the first manifestation of disease; therefore, our study may be susceptible to survival bias. However, this is common to many clinical studies, and our referral base was otherwise broad and representative of the population. Second, there were differences in sex and ethnicity between London cases and controls. However, myocarditis is known to have a 2:1 male preponderance, and our cases were shown to match the demographics of national admission data, which highlighted that men are typically ≈15 years younger than women at first clinical presentation.^24^ This supports earlier findings from a smaller nationwide registry of 3198 patients in Finland.^45^ The reasons for this difference are complex, but sex may be a third factor that alters the threshold at which an environmental trigger in a genetically predisposed individual causes disease: a triple hit. The greater ethnic diversity among controls should, if anything, contribute to greater genetic variation in these controls compared with cases. However, sensitivity analyses confirmed that the key findings were not affected by ethnicity and remained significant when assessed against the 141 456 unrelated individuals in gnomAD. Third, although we selected genes that were robustly associated with cardiomyopathy, we did not include *FLNC*, which has recently gained interest as a result of involvement in the pathogenesis of left-dominant forms of ACM.^46^ We are seeking to evaluate this in our cohort. Last, our diagnoses were based on CMR or biopsy confirmation, although future studies may also seek to include novel circulating microRNAs to refine diagnoses or to facilitate recruitment of larger study cohorts.^47^ Such future studies may explore the potential impact of family history on the selection of patients with myocarditis for genetic sequencing.

### Conclusions

One in 13 cases with acute myocarditis harbored an underlying pathogenic or likely pathogenic variant in key cardiomyopathy-associated genes. This highlights the potential role of genetic sequencing in patients presenting with acute myocarditis and supports the concept that genotype-positive individuals may remain phenotypically silent until the occurrence of an environmental trigger. Further studies are required to dissect the mechanisms to help target therapy and to provide greater understanding of how genetic variants influence long-term clinical outcomes. With the increasing prevalence of myocarditis secondary to immune checkpoint inhibitors and both coronavirus disease 2019 (COVID-19) infection and vaccination, our findings may additionally provide understanding to guide susceptibility and risk stratification in these areas.

## Article Information

### Acknowledgments

The authors thank all their colleagues across Northwest London and Maastricht for their help in identifying and referring study participants in the acute setting. The authors also thank NHS Digital for curating, extracting and supplying the relevant national hospital admission data (https://digital.nhs.uk/). A.S.L., J.S.W., and S.K.P. were responsible for the conception and design of the study. A.S.L., R.W., and S.S. coordinated and completed the study recruitment visits in London. A.S.L., R.B., P.T., and J.S.W. were responsible for performing and interpreting genetic sequencing in London. M.R.H., J.V., and S.H. coordinated and completed the study recruitment in Maastricht and performed the genetic sequencing. V.V. was responsible for adjudicating CMR data in London. R.E.J. and D.J.H. were responsible for adjudicating all clinical outcomes. A.S.L., D.M., and I.T. were responsible for analyzing national NHS admission data. A.S.L., P.T., M.R.H., R.O., J.S.W., and S.K.P. were responsible for data analysis and had access to all the study data. A.S.L. drafted the manuscript with critical review and contribution to interpretation of the data from all authors, including the decision to submit the manuscript. The views expressed in this work are those of the authors and not necessarily those of the funders.

### Sources of Funding

The study was funded and supported by Alexander Jansons Myocarditis UK, British Heart Foundation (FS/17/21/32712 awarded to Drs Lota and Prasad; RE/18/4/34215; FS/ICRF/21/26019 awarded to Dr Halliday), Cardiovascular Research Centre at the Royal Brompton and Harefield Hospitals, Medical Research Council (UK), National Institute for Health Research Royal Brompton Cardiovascular Biomedical Research Unit, National Institute for Health Research Imperial College Biomedical Research Centre, National Heart and Lung Institute Foundation, Royston Centre for Cardiomyopathy Research, the Wellcome Trust, Foundation Leducq, and the Instituto de Salud Carlos III. Dr Prasad has received research grant funding from the Alexander Jansons Foundation, Rosetree Trust, British Heart Foundation, Medical Research Council, and Coronary Artery Disease Research Association. Dr Hazebroek has received funding from the Kootstra Talented Post-Doc Fellowship. Dr Heymans acknowledges support from the Netherlands Cardiovascular Research Initiative, an initiative with support of the Dutch Heart Foundation; Dutch Cardiovascular Alliance; CVON Arena-PRIME, 2017-18 for gene sequencing; and Double Dosis 2020-B005 for patient inclusion. The Centro Nacional de Investigaciones Cardiovasculares (CNIC) is supported by the Instituto de Salud Carlos III, Ministerio de Ciencia, the Pro-CNIC Foundation, and the Severo Ochoa Centers of Excellence program (CEX2020-001041-S).

### Disclosures

Dr Prasad has received honoraria from Bayer Schering and travel support from Circle 42, outside the submitted work. Dr Ware has received personal fees from Myokardia and Foresite Labs, outside the submitted work. Dr Heymans receives personal fees for scientific advice to AstraZeneca, Cellprothera, and CSL Behring and an unrestricted research grant from Pfizer. Dr Pennell has received research support from Siemens and Bayer and personal fees from Bayer and Chiesi, outside the submitted work. The other authors report no conflicts.

### Supplemental Material

List of investigators

Geographical catchment area of study recruitment

Burden of myocarditis admissions across North West London

Expanded Methods: CMR protocol; DNA extraction, sequencing, bioinformatics, and quality control

Detailed cohort demographics

Detailed genetic findings

Additional phenotypic data

Figures S1–17

Tables S1–S9

References 48–50

## Supplementary Material


