# Supplementary material for: Genetic Architecture of Acute Myocarditis and the Overlap With Inherited Cardiomyopathy
Source: Circulation. 2022 Sep 26;146(15):1123–34. doi: 10.1161/CIRCULATIONAHA.121.058457 (PMC9555763; doi:10.1161/CIRCULATIONAHA.121.058457)

Supplementary Material

Contents page

List of investigators....2

Geographical catchment area of study recruitment....3

Burden of myocarditis admissions across North West London in 2017 and 2018....4

CMR Protocol....7

DNA extraction and targeted sequencing in London....7

Bioinformatics and quality control....8

Evaluation of variant quality by Ti/Tv ratio....9

Evaluation of depth of sequencing coverage....11

Genetic ethnicity of myocarditis cases and healthy volunteers....12

Myocarditis cohort in Maastricht – genetic sequencing method....13

Detailed cohort demographics....14

Detailed genetic findings....15

Additional phenotypic data....24

List of investigators

Listed in alphabetical order by patient identification centre:

Barts Hospital (Dr Sam Mohiddin), Basildon Hospital (Dr Jason Dungu), Chelsea & Westminster Hospital (Dr Sam Kaddoura, Dr Julian Collinson, Dr Resham Baruah), Conquest Hospital (Dr Andrew Marshall), Ealing Hospital (Dr Ravi Assomull), Hammersmith Hospital (Dr Nilesh Sutaria, Dr Declan O'Regan), Harefield Hospital (Dr Nick Banner, Dr Owais Dar, Dr Tito Kabir), Hillingdon Hospital (Dr Simon Dubrey), Kingston Hospital (Dr Arvind Vasudeva), Lister Hospital (Dr Paul Kirk), North Middlesex Hospital (Dr Amal Muthumala, Dr Roger Rear), Northwick Park Hospital (Dr Nigel Stephens, Dr Jaymin Shah, Dr Andonis Violaris, Dr Hugh Bethell), Milton Keynes University Hospital (Dr Cliona Kenny), Royal Brompton Hospital (Dr Francisco Alpendurada, Dr Cemil Izgi, Dr Sabiha Gati, Dr Raad Mohiaddin), Watford Hospital (Dr Niall Keenan), West Middlesex Hospital (Dr Sadia Khan), West Suffolk Hospital (Dr Vassilis Vassillou), Wexham Park Hospital (Dr Dinos Missouris, Dr Nav Chalal), William Harvey Hospital (Dr Paula Mota, Dr Paul Fenton) & Queen Elizabeth Hospital Woolwich (Dr Carl Shakespeare).

Geographical catchment area of study recruitment in London and Maastricht

Patients with acute myocarditis were recruited between 17th June 2016 – 18th June 2018 from regional and tertiary medical centres across 9 London boroughs in North West London. The total population of this region was recorded as 2.3 million people in all age groups by the Office of National Statistics in 2018. Patients with acute myocarditis were recruited from 2 Dutch provinces in the Netherlands over a similar period with a regional population of 3.6 million people according to national statistics.

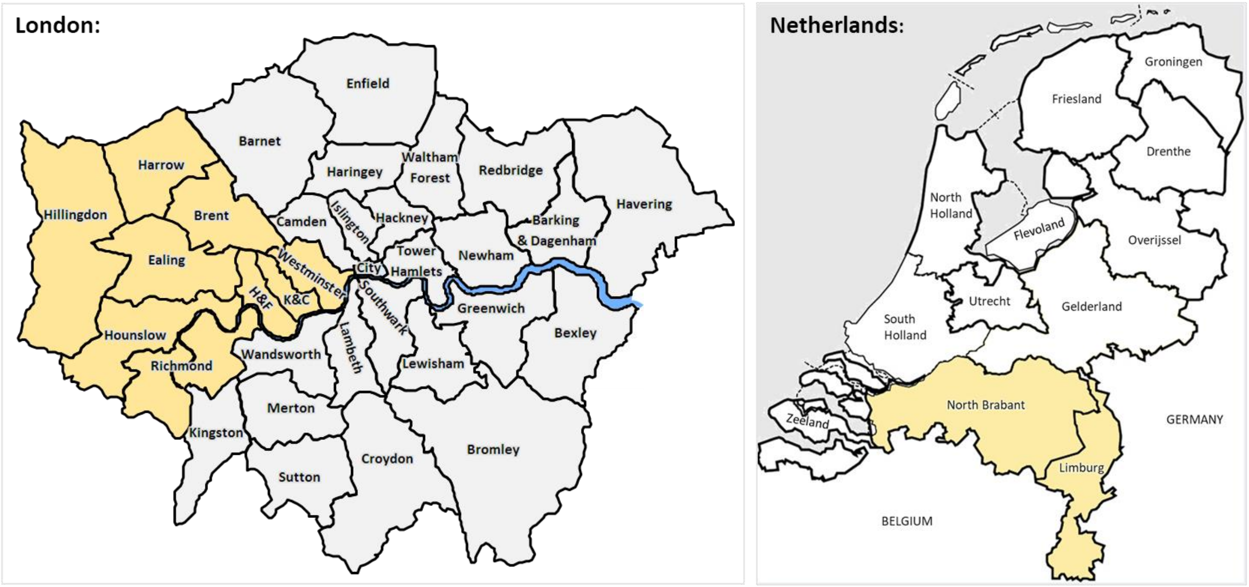

Supplementary Figure 1. Maps of London (UK) and Maastricht (Netherlands) showing the study recruitment catchment areas (highlighted in yellow) with a population of 2.3 million in North West London and 3.6 million in 2 Dutch provinces

Burden of myocarditis admissions across North West London in 2017 and 2018

The numbers of acute myocarditis admissions across the London boroughs were extracted from the Global Burden of Disease 2019 subnational dataset in order to assess; (i) regional differences in disease prevalence, and (ii) temporal differences between the two main years of study recruitment.

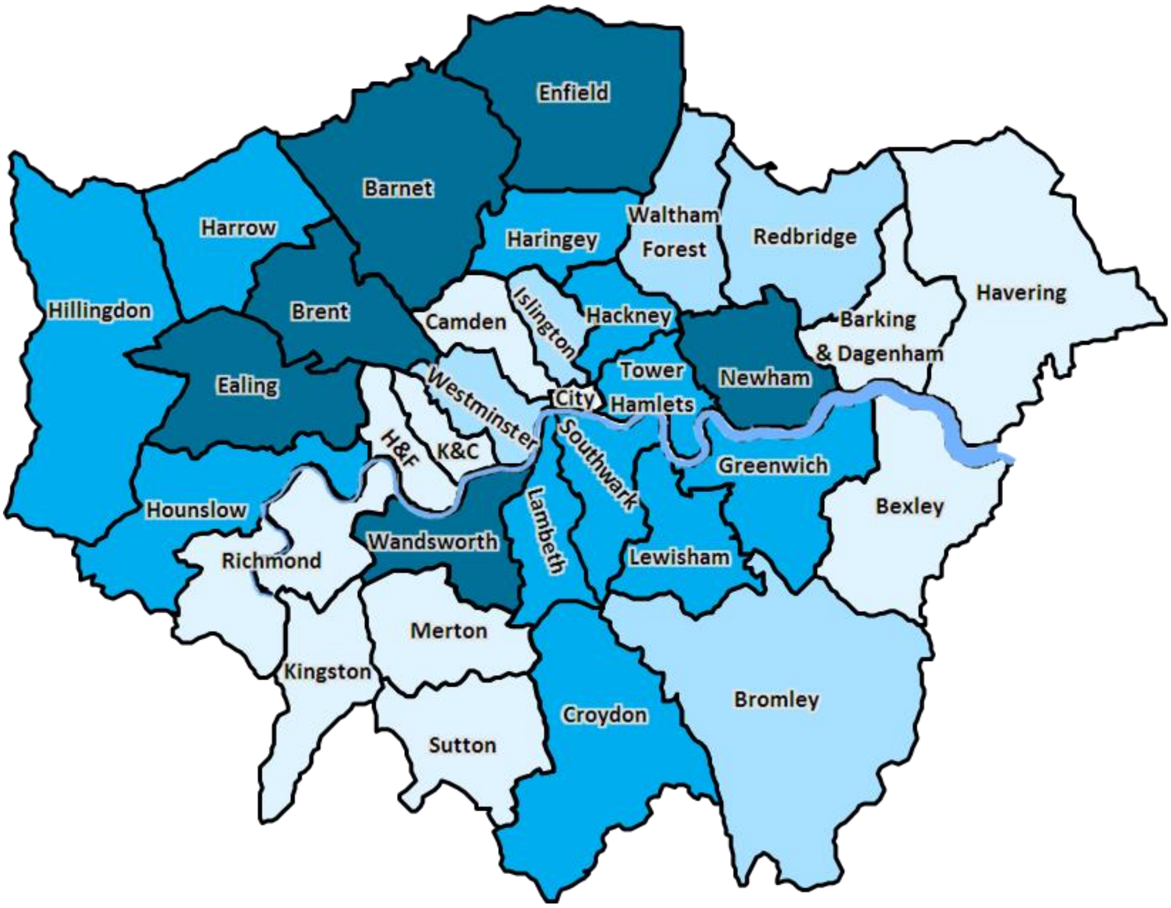

	2017 year (n=599)			2018 year (n=604)		
Equal ranges	Lower limit	Upper limit	Boroughs	Lower limit	Upper limit	Boroughs
1	10.7	17.1	11	10.6	17.0	11
2	17.1	23.5	4	17.0	23.5	4
3	23.5	29.9	12	23.5	30.0	12
4	29.9	36.3	5	30.0	36.5	5
No data*			1			1

Supplementary Figure 2. Heat map showing number of admissions due to acute myocarditis divided into four equal groups across all boroughs in London between ages 15-69 years in the 2017 and 2018 calendar years based on the Global Burden of Disease 2019 dataset. * Data on the City was not available due to the small resident population (~8000 people).

Based on these analyses, admissions due to myocarditis were greatest in the North West of London and there was no significant change in the number or the regional distribution of cases across the two years of study recruitment. This regional difference was not attributed to population density and may relate to other confounding factors.

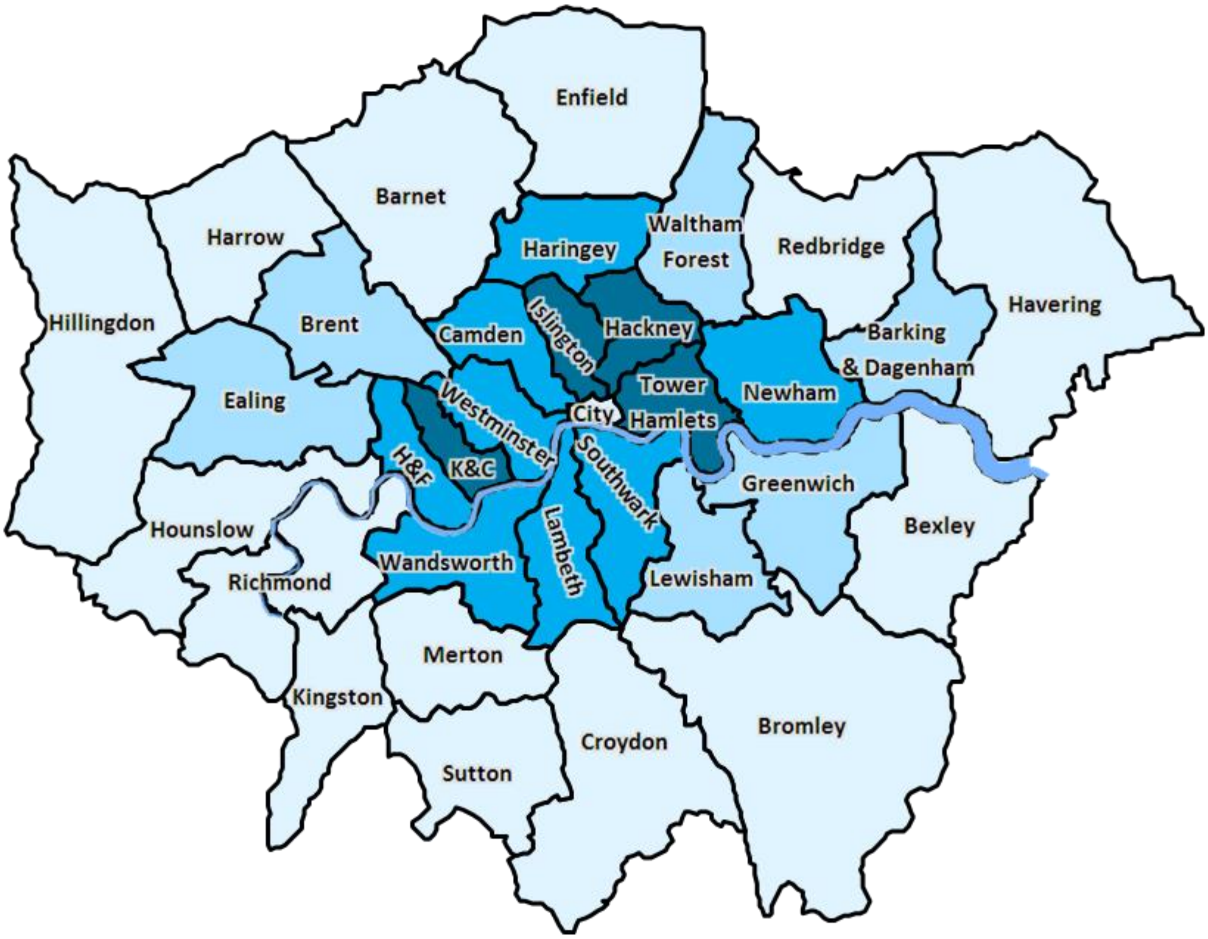

Supplementary Figure 3. Heat map showing population density divided into four equal groups across all boroughs in London 9 years in the 2018 calendar years based on the Office of National Statistics (ONS) Data.

Further exploration of the age distribution of cases across all 33 boroughs demonstrated a peak incidence between 30-34 years, which also remained stable in both years of recruitment. This matched what was observed within our recruited myocarditis cohort, again suggesting it was representative of the cases seen across London. However, data on sex distribution was

not available from GBD data and hence further exploration using NHS Digital hospital episode statistics admitted patient care data.

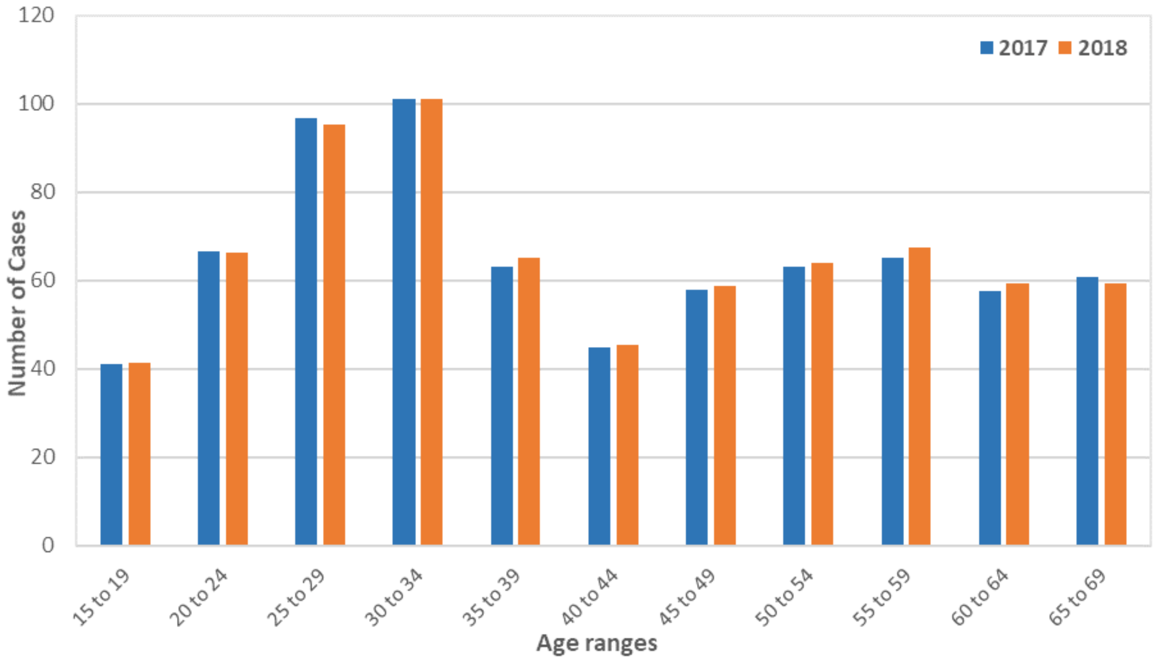

Supplementary Figure 4. Bar chart to show age distribution of patients with acute myocarditis in 2017 and 2018 across all 33 boroughs of London from GBD 2019

CMR Protocol

T2-weighted sequences with short-tau inversion recovery (T2-STIR) were acquired to evaluate the presence of myocardial oedema. Gadolinium contrast agent (0.1mmol/kg) of either Magnevist or Gadovist (Bayer) was used with inversion-recovery gradient echo sequences to evaluate the presence of late gadolinium enhancement. For both sequences, images were repeated in two separate phase-encoding directions in multiple orthogonal views to exclude artefacts. Left ventricular volumes, ejection fraction, and mass were measured using dedicated software (CMRtools) and indexed to body surface area. Blood pool thresholding was used to delineate and exclude the papillary muscles from ventricular volumes. CMR was deemed positive for myocarditis when myocardial oedema and mid-wall/subepicardial LGE were both present. The presence of early gadolinium enhancement provided supporting information but the ratio with skeletal muscle was not routinely assessed. Healthy volunteers did not receive gadolinium contrast.

DNA extraction and Targeted sequencing in London

DNA extraction was performed from whole blood (single 0.5mL cryovial) using an automated platform (EZ1 Advanced XL, Qiagen). DNA quantity and quality was assessed using nanodrop (Thermo Scientific). For library preparation, specific hybridisation capture probes were used to target and enrich the selected genes implicated in cardiac conditions using the TruSight Cardio kit (Illumina). This included 169 genes associated with inherited cardiac conditions. The biotin/streptavidin pull down method was used, where rapid capture oligos specifically bound to DNA of the target genes were captured with streptavidin coated magnetic beads and then amplified with 12 cycles of PCR with indexing primers. Following elution from the magnetic beads, the targeted DNA sequencing library was then amplified ('solid-phase bridge amplification') again for 16hours on the Illumina flow cell to generate clusters of primed DNA. Sequencing was performed on the Illumina NextSeq platform

generating 100-150bp reads. Healthy volunteer samples were prepared and sequenced using the same approach.

Bioinformatics and Quality Control

Variants were annotated and filtered using a well-validated bioinformatics pipeline. In brief, NGS reads underwent quality control per sample before being aligned against a reference human genome (hg19) using the Burrows-Wheeler Aligner algorithm (BWA-0.7.17). Aligned reads were pre-processed to identify any duplicates and recalibrate quality scores using the genome analysis toolkit (GATK version 4.1.9.0). The GATK HaplotypeCaller was used for variant calling, followed by joint-genotyping and variant filtering using GATK best practices. The Ensembl Variant Effects Predictor (VEP version 103) was used for annotation of genomic consequence. The annotated multi-sample VCF was taken forward for quality control and burden testing analysis. The frequencies of rare variants were evaluated in cases against healthy controls using minor allele frequency $<1.0e^{-4}$ and population data in gnomAD with stringent filtering of population allele frequencies (using Popmax filtering AF (95% confidence)).

Supplementary Table 1. DCM and ACM genes included in the pre-specified in the primary analysis. Variant classes are also listed.

	Gene symbol	Non-truncating	Truncating	Transcript ID
**DCM**	*BAG3*	No	Yes	ENST00000369085
	DES	Yes	Yes	ENST00000373960
	LMNA	Yes	Yes	ENST00000368300
	MYH7	Yes	No	ENST00000355349
	PLN	Yes	Yes	ENST00000357525
	RBM20	Yes	Yes	ENST00000369519
	SCN5A	Yes	Yes	ENST00000413689
	TNNCI	Yes	No	ENST00000232975
	TNNT2	Yes	No	ENST00000509001
	TPM1	Yes	No	ENST00000358278
	TTN	No	Yes*	ENST00000589042
**ACM**	*DSC2*	Yes	Yes	ENST00000280904
	DSG2	Yes	Yes	ENST00000261590
	DSP	Yes	Yes	ENST00000379802
	JUP	Yes	Yes	ENST00000393931
	PKP2	No	Yes	ENST00000070846

The genes assessed in this study are those with a demonstrated excess of rare variation in DCM and ACM clinical cohorts over reference databases with definitive or strong evidence of disease association for the variant classes listed above.

*Titin truncating variants (TTN-tv) were only included if identified in constitutively expressed exons with percentage spliced in (PSI) >0.9.

Evaluation of variant quality by Ti/Tv ratio

The mean Ti/Tv ratios for the myocarditis and healthy volunteer's cohorts were 2.85 and 2.81, respectively (p=not significant). These fall within the expected ranges using GATK.

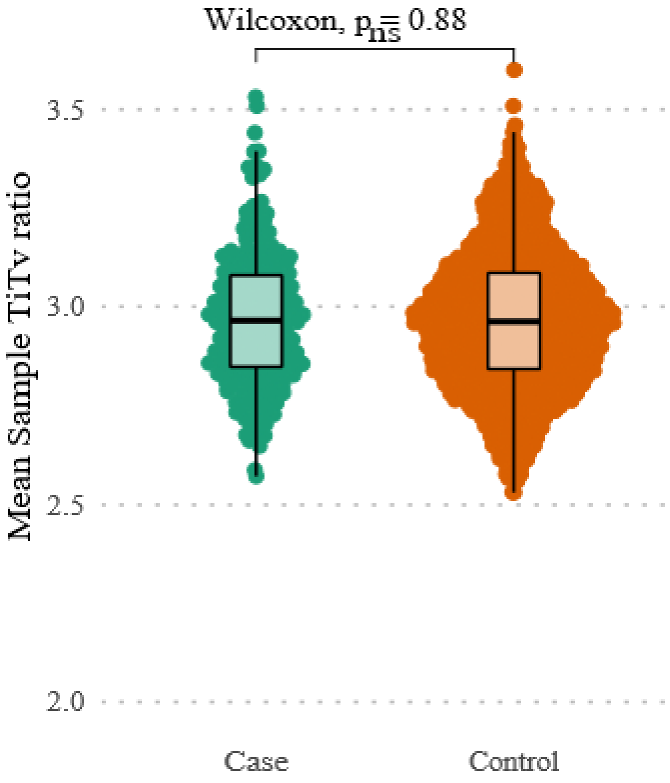

Supplementary Figure 6. Bee swarm plot showing transition-to-transversion ratio of myocarditis cases (mean 2.85) and healthy controls (mean 2.81). [HC = Haplotype Caller]

Evaluation of depth of sequencing coverage

All samples in the myocarditis cohort were covered at read depth ≥30x and 96.5% were covered at ≥50x indicating the accuracy of genome alignment algorithms. The median coverage was approximately 700x in both cohorts on our platform.

Supplementary Table 2. Summary of median coverage depth in myocarditis cases vs HVols.

		Bases >=10x	Bases 20x	Bases >=30x	Bases >=50x
**Myocarditis**	Median	99.98	99.94	99.88	99.69
	1st quartile	99.94	99.88	99.78	99.48
	3rd quartile	99.99	99.96	99.93	99.86
**Hvols**	Median	99.97	99.93	99.89	99.74
	1st quartile	99.95	99.89	99.80	99.48
	3rd quartile	99.99	99.96	99.93	99.84

Supplementary Figure 7. Bee swarm plot showing all individual healthy control (cohort 2) and myocarditis (cohort 1) samples with read depth >= 20x (left) and mean coverage (right).

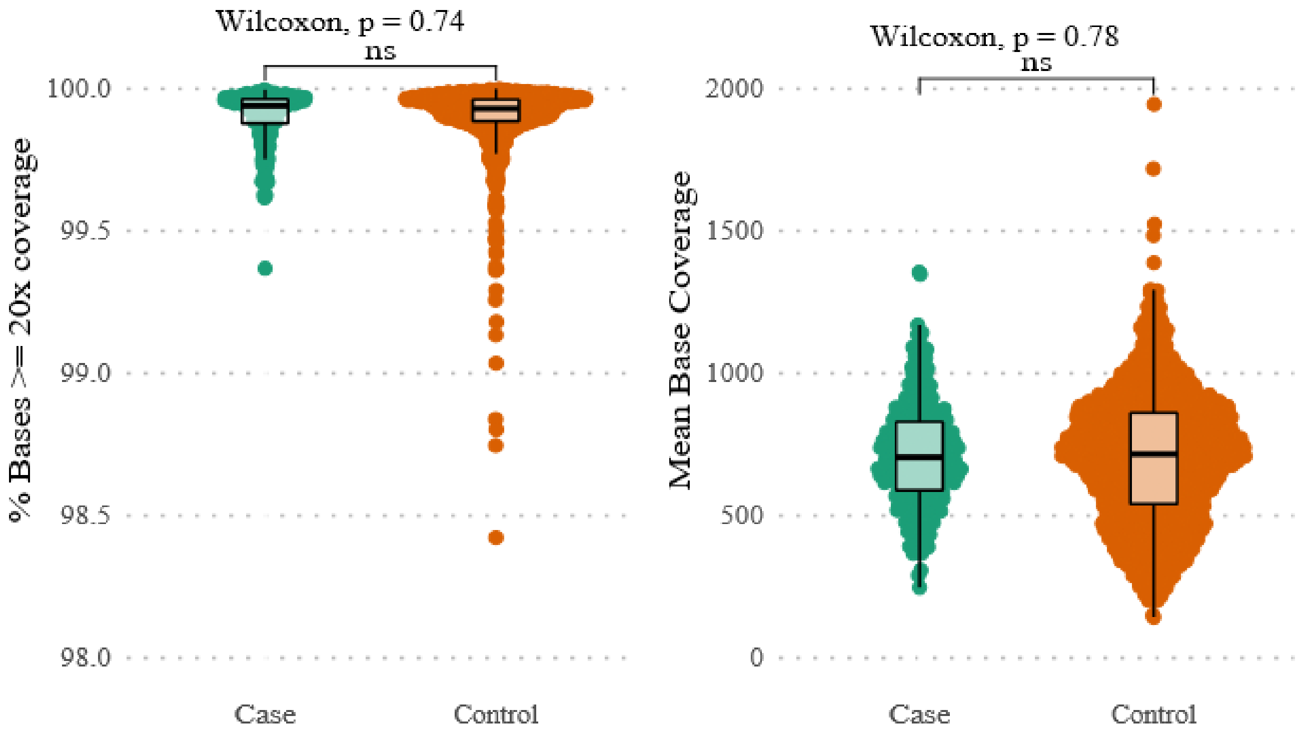

Genetic ethnicity of myocarditis cases and healthy volunteers

In addition to self-reported ethnicity, we calculated the genetic ethnicity of myocarditis cases and healthy volunteers using principal component analysis (PCA) of sequencing data and compared this with an open-access reference dataset, HapMap3.

Sensitivity analysis confirmed that ethnicity did not influence the key findings, as the genetic variants assigned as likely pathogenic or pathogenic in the London cohort were all reported in Caucasian individuals with the exception of a single Afro-Caribbean patient with a truncating variant in *TTN*.

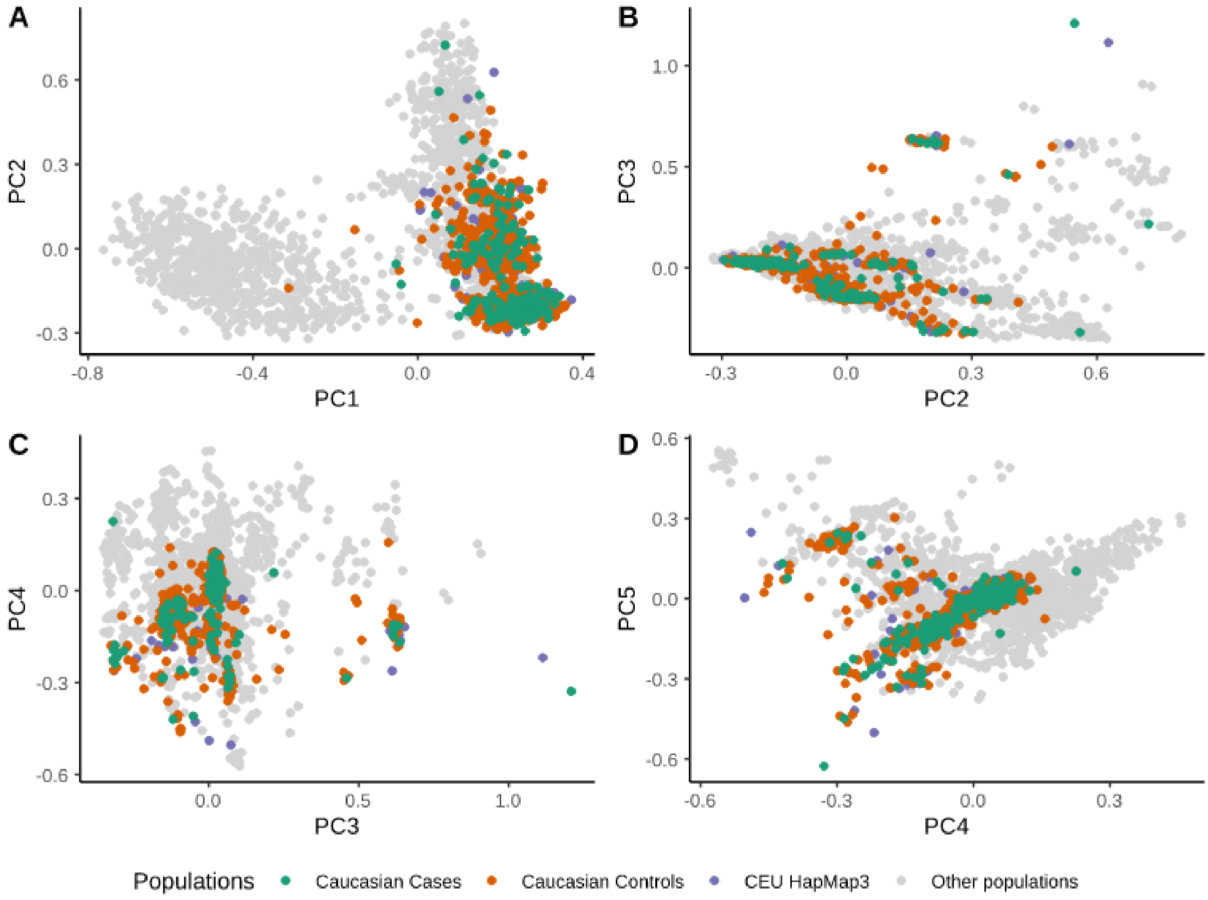

Supplementary Figure 8. Principal component analyses (PCA) on the Hardy-Weinberg-normalized genotype call matrix of all European-ancestry myocarditis cases (cohort 1) and healthy volunteers (cohort 2) compared with Europeans in HapMap confirming good agreement between self-reported ethnicity and HapMap (Panels A-D represent the relationships between the top 5 principal components).

Myocarditis Cohort in Maastricht – Genetic Sequencing Method

The pre-specified DCM-associated genes were analyzed using NGS, either with whole exome sequencing (WES - SureSelectXT Human All Exon V4+UTRs target capture coupled with Illumina Hiseq2000 sequencing), or with a panel of 47 genes captured using single molecule Molecular Inversion Probe (smMIP) enrichment followed by multiplexed analysis on an Illuimna NextSeq 500 System[48,49]. In both cases the same 47 DCM-associated genes were initially interrogated. All variants identified were validated with Sanger sequencing. Variants were classified by standard terminology in 5 different classes: pathogenic, likely pathogenic, variant of clinical unknown significance (VUS), likely benign or benign. Classification of variants was based on the score of in silico prediction software scores (SIFT, MutationTaster, PolyPhen-2, PhyloP, Align-GVGD), the frequency in reference population databases (ExAC, gnomAD, 1000 genomes, ESP projects) and previously published variations in NCBI's ClinVar and HGMD. Both pathogenic and likely pathogenic mutations were classified as pathogenic variants. All others were considered as non-pathogenic based on the current knowledge. Titin variants were only considered pathogenic in case of truncating variants in the late I-band or A-band region with percentage spliced in (PSI) >99%.[50]

Detailed Cohort Demographics

Supplementary Table 3. Baseline demographics of all acute myocarditis patients in cohort 1 (London) at the time of study recruitment

	**All patients** (n=230)	**Prospective** (n=114)	**Retrospective** (n=116)	**P value**
Median Age (IQR), years	35 (27-47)	31 (25-40)	40 (28-53)	0.258
Male, n (%)	193 (84)	96 (84)	97 (83)	0.860
Caucasian	203 (88)	96 (84)	107 (91)	0.108
Recent viral illness	197 (85)	93 (81)	104 (89)	0.139
Recent coryzal symptoms	86 (37)	46 (40)	40 (34)	0.344
Recent gastrointestinal upset	35 (18)	20 (18)	15 (47)	0.361
Previous history of myocarditis	11 (5)	4 (4)	7 (6)	0.539
Family history of myocarditis	8 (3)	3 (3)	5 (4)	0.722
Family history of ACM / DCM	1 (0)	0 (0)	1 (1)	1
Excess alcohol	37 (16)	18 (16)	19 (16)	1
Clinical presentation				
Chest pain	196 (85)	101 (89)	95 (82)	0.104
Breathlessness	97 (42)	49 (43)	48 (41)	0.791
Palpitations	42 (18)	21 (18)	28 (24)	0.337
Syncope	26 (11)	11 (10)	15 (13)	0.533
Median interval symptom onset to FMC (IQR), days	1 (0-3)	1 (0-3)	1 (0-3)	0.797
Median interval symptom onset to admission	1 (0-3)	1 (0-3)	1 (0-4)	0.096
Median duration of index hospital admission	4 (2-6)	4 (2-6)	3 (1-6)	0.275
Investigations (at time of recruitment)				
ECG ST or Tw changes, n (%)	144 (63)	93 (82)	51 (44)	<0.0001
ECG arrhythmia	27 (12)	19 (17)	8 (7)	0.024
Troponin elevation	117 (51)	109 (96)	8 (7)	<0.0001
BNP elevation	94 (41)	61 (54)	33 (28)	<0.0001
Evidence of viral pathogen	35 (22)	35 (31)	not assessed	-
Endocardial biopsy or surgical excision of cardiac tissue	11 (5)	6 (5)	5 (4)	0.767
Giant cell myocarditis	4 (2)	2 (2)	2 (2)	1.000
CMR parameters				
Median interval first medical contact to baseline CMR (IQR), days	7 (4-15)	6 (3-12)	9 (4-27)	0.016
LVEDVi, ml/m2	87 (74-102)	87 (76-102)	82 (74-102)	0.438
LVESVi, ml/m2	32 (26-40)	32 (27-39)	31 (26-41)	0.583
LVEF, %	63 (57-67)	63 (57-67)	63 (58-67)	0.927
LV mass index, g/m2	72 (62-85)	75 (65-87)	66 (57-58)	0.012
RVEDVi, ml/m2	90 (77-107)	89 (77-106)	96 (80-112)	0.509
RVESVi, ml/m2	38 (33-48)	38 (33-48)	41 (33-49)	0.963
RVEF, %	57 (51-61)	57 (51-61)	58 (52-60)	0.482

Detailed Genetic Findings

Supplementary Figure 9. Excess burden of rare variants in London myocarditis cases (cohort 1) vs healthy controls (cohort 2) in the key ACM and DCM genes. In aggregate, truncating variants in ACM genes were significantly enriched in myocarditis cases [truncating variants present in 3% cases vs 0% controls, Δ+3%, odds ratio 8.2 (confidence interval 2.4-28.3), Pfisher< 0.001]. Of these, *DSP* truncating variants were the most significant (OR 27.8; 95% CI 1.4-557.6; Pfisher=0.006; indicated as ** below). Truncating variants in DCM genes were not significantly enriched in myocarditis cases.

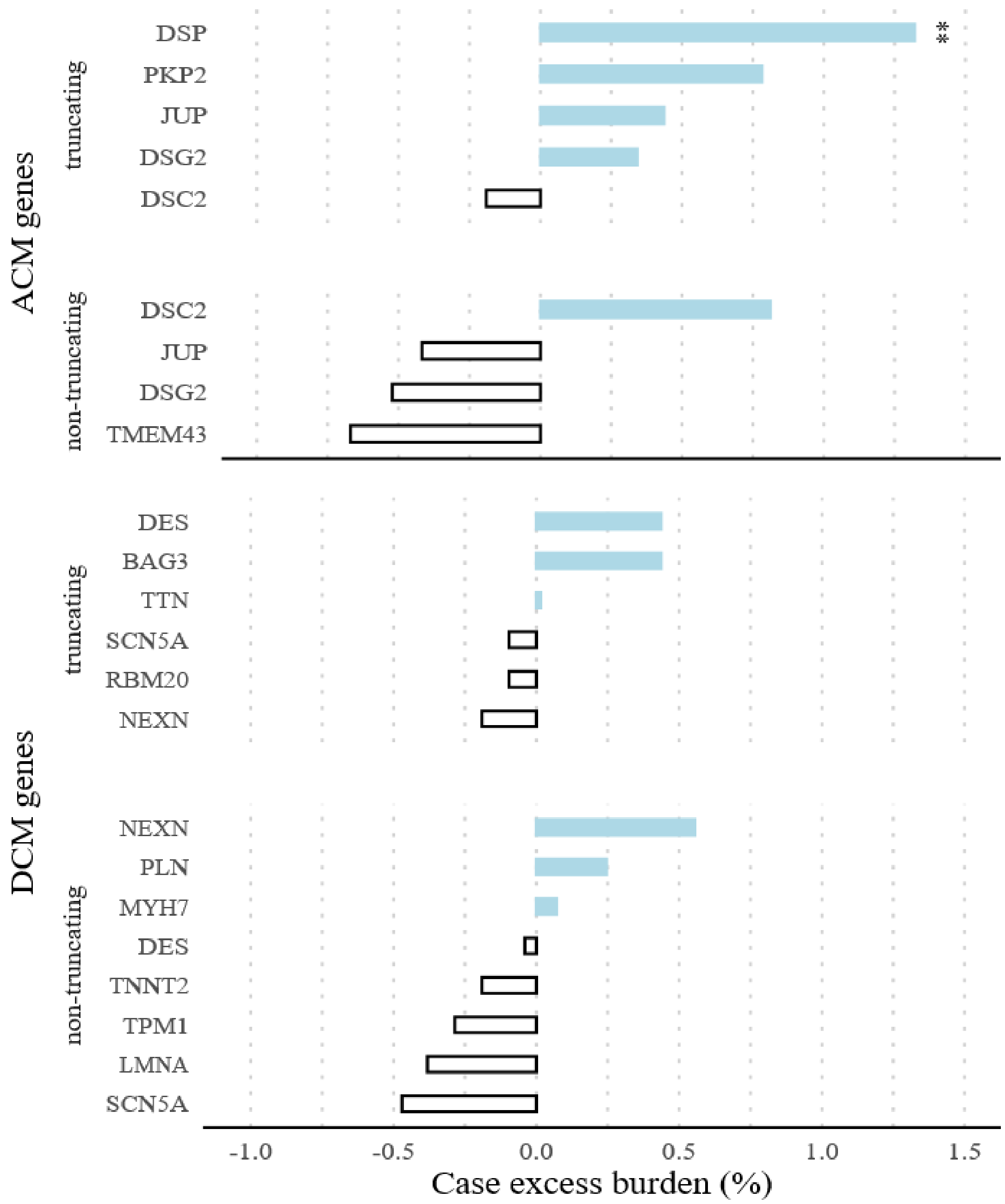

Supplementary Table 4. Odds ratios and Fisher's Exact test results testing for significance of the excess of rare truncating variation in myocarditis cases (cohort 1) versus controls in ACM genes (Fisher's Exact test 2-sided level of significance = 0.05, with Bonferroni correction for 5 tests = 0.01). For cells with zero values, 0.5 was added to all cells before calculating the odds ratio (highlighted in green).

Gene	Variant Class	Cases +ve	Cases -ve	Controls +ve	Controls -ve	Case freq	Control freq	Odds ratio	CI lower	CI upper	Fishers exact
DSC2	Truncating	0	230	2	1051	0.000	0.002	1.1	0.1	25.4	1.000
DSG2	Truncating	1	229	1	1052	0.004	0.001	4.6	0.3	73.7	0.327
DSP	Truncating	3	227	0	1053	0.013	0.000	27.8	1.4	557.6	0.006
JUP	Truncating	1	229	0	1053	0.004	0.000	9.2	0.3	275.0	0.179
PKP2	Truncating	2	228	1	1052	0.009	0.001	9.2	0.8	102.2	0.085
All	Truncating	7	223	4	1049	0.031	0.004	8.2	2.4	28.4	0.001
All (except DSP)	Truncating	4	226	4	1049	0.018	0.004	4.6	1.2	18.7	0.039

Supplementary Table 5. Odds ratios and Fisher's Exact test results testing for significance of the excess of rare truncating variation in myocarditis cases (cohort 1) versus controls in DCM genes (Fisher's Exact test 2-sided level of significance = 0.05, with Bonferroni correction for 11 tests = 0.005). For cells with zero values, 0.5 was added to all cells before calculating the odds ratio (highlighted in green).

Gene	Variant Class	Cases +	Cases -	Controls +	Controls -	Case freq	Control freq	Odds ratio	CI lower	CI upper	Fishers exact
BAG3	Truncating	1	229	0	1053	0.004	0.000	9.2	0.3	275.0	0.180
LMNA	Truncating	0	230	0	1053	0.000	0.000	-			
MYH7	Truncating	0	230	3	1050	0.000	0.003	0.8	0.0	15.2	1
RMB20	Truncating	0	230	1	1052	0.000	0.001	2.3	0.1	68.4	1
SCN5A	Truncating	0	230	1	1052	0.000	0.001	2.3	0.1	68.4	1
TCAP	Truncating	0	230	0	1053	0.000	0.000	-			
TNNC1	Truncating	0	230	0	1053	0.000	0.000	-			
TNNT2	Truncating	0	230	0	1053	0.000	0.000	-			
TPM1	Truncating	0	230	1	1052	0.000	0.001	2.3	0.1	68.4	1
TTN	Truncating	3	227	9	1044	0.013	0.009	1.5	0.4	5.7	0.459
VCL	Truncating	0	230	0	1053	0.000	0.000	-			
All	Truncating	4	226	15	1038	0.017	0.014	1.2	0.4	3.7	0.762

Supplementary Table 6. Odds ratios and Fisher's Exact test results testing for significance of the excess of rare variation in myocarditis cases (cohort 1) versus gnomAD controls in ACM genes (Fisher's Exact test 2-sided level of significance = 0.05, with Bonferroni correction for 5 tests = 0.01). For cells with zero values, 0.5 was added to all cells before calculating the odds ratio (highlighted in green).

Gene	Variant Class	Cases +	Cases -	Controls +	Controls -	Case freq	Control freq	Odds ratio	CI lower	CI upper	Fishers exact
DSC2	Truncating	0	230	52	141404	0.0000	0.0004	5.9	0.4	96.1	1
DSG2	Truncating	1	229	87	141369	0.0043	0.0006	7.1	1.0	51.2	0.129
DSP	Truncating	3	227	112	141344	0.0130	0.0008	16.7	5.3	52.9	0.001
JUP	Truncating	1	229	16	141440	0.0043	0.0001	38.6	5.1	292.3	0.027
PKP2	Truncating	2	228	101	141355	0.0087	0.0007	12.3	3.0	50.1	0.012
All	Truncating	7	223	368	141088	0.0304	0.0026	12.0	5.6	25.7	<0.00001

Supplementary Figure 10. All protein-altering variants in the London cohort according to prospective or retrospective recruitment. In aggregate, the prospective case frequency of DCM-tv was 0.008 and ACM-tv was 0.025, versus retrospective case frequencies of 0.026 and 0.035, respectively (p=ns).

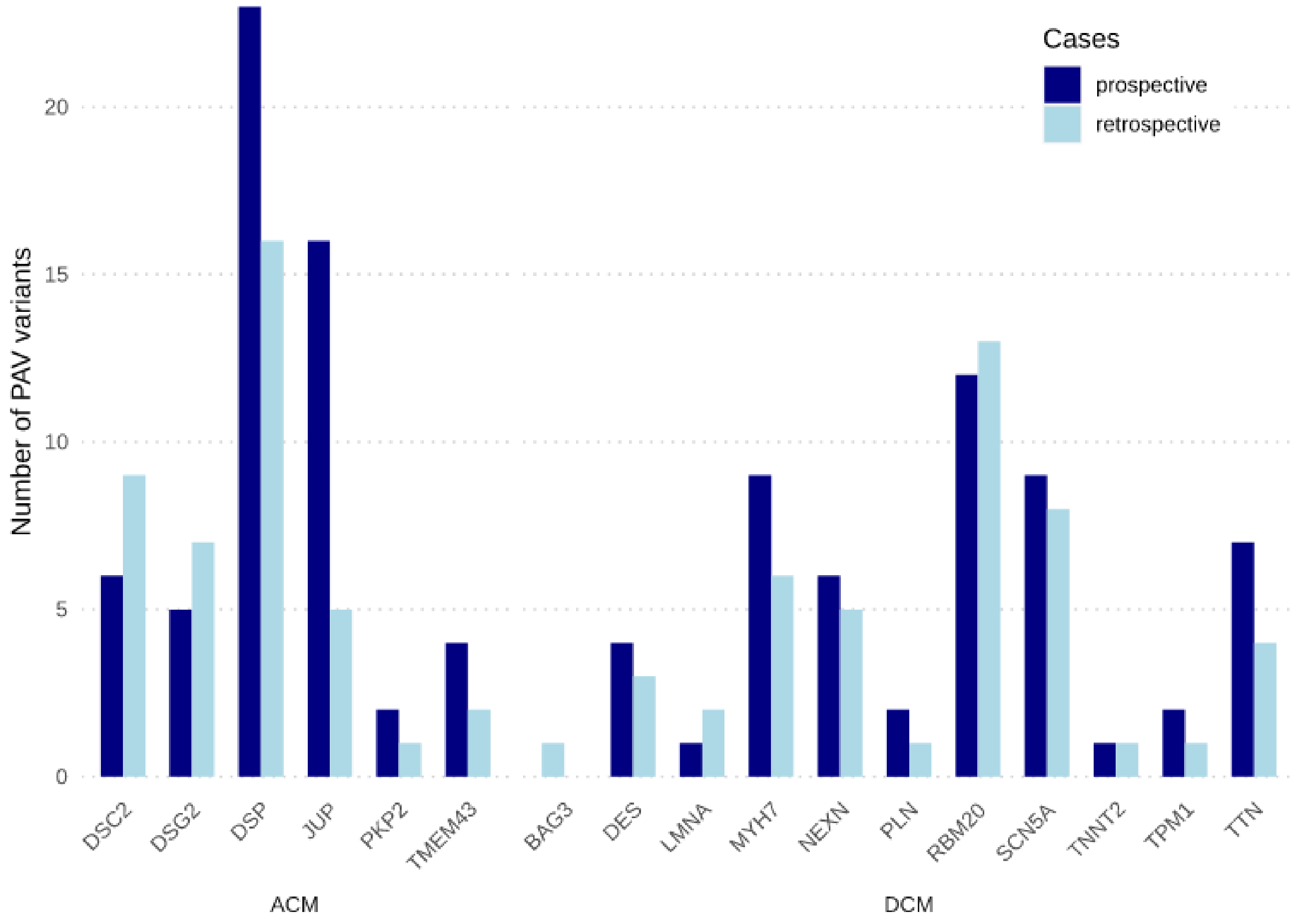

Supplementary Table 7. All rare variants and protein consequences identified in the London cohort of myocarditis cohort (n=230)

Gene	HGVSc	Protein	ACMG class
BAG3	c.235del	p.Ala79LeufsTer132	Likely Pathogenic
DES	c.1048C>T	p.Arg350Trp	Likely Pathogenic
DSG2	c.829_840del	p.Leu277_Met280del	Likely Pathogenic
DSP	c.4307_4308del	p.Thr1436ArgfsTer3	Likely Pathogenic
DSP	c.4423del	p.Thr1475ProfsTer9	Likely Pathogenic
DSP	c.5056C>T	p.Q1686X	Likely Pathogenic
PKP2 [1]	c.968_969del	p.Gln323ArgfsTer12	Likely Pathogenic
PKP2	c.337-2A>T		Likely Pathogenic
TTN	c.90688G>T	p.G30230X	Likely Pathogenic
TTN	c.51459_51462del	p.Asp17153GlufsTer11	Likely Pathogenic
DSC2	c.1559T>C	p.Ile520Thr	Uncertain Significance
DSC2	c.835C>T	p.Arg279Cys	Uncertain Significance
DSC2	c.173T>A	p.Phe58Tyr	Uncertain Significance
DSC2	c.82G>T	p.Ala28Ser	Uncertain Significance
DSG2	c.1038_1040del	p.Lys346del	Uncertain Significance
DSP	c.1351C>G	p.Arg451Gly	Uncertain Significance
DSP	c.3099G>C	p.Lys1033Asn	Uncertain Significance
DSP	c.8500C>T	p.Arg2834Cys	Uncertain Significance
JUP	c.1797del	p.Asn599LysfsTer88	Uncertain Significance
JUP	c.1359G>T	p.Glu453Asp	Uncertain Significance
JUP	c.682A>G	p.Ile228Val	Uncertain Significance
JUP [2]	c.344G>A	p.Arg115Gln	Uncertain Significance
JUP	c.*14+4C>T		Uncertain Significance
MYH7	c.5243G>A	p.Cys1748Tyr	Uncertain Significance
MYH7 [1]	c.2585C>T	p.Ala862Val	Uncertain Significance
MYH7 [3]	c.1045A>G	p.Met349Val	Uncertain Significance
MYH7	c.4169+5G>A		Uncertain Significance
PLN	c.61C>A	p.Pro21Thr	Uncertain Significance
RBM20 [3]	c.1741A>G	p.Ile581Val	Uncertain Significance
RBM20	c.2183_2185del	p.Glu728del	Uncertain Significance
SCN5A	c.2815C>T	p.Leu939Phe	Uncertain Significance
SCN5A [2]	c.1442G>A	p.Arg481Gln	Uncertain Significance
SCN5A	c.3666+7T>A		Uncertain Significance
SCN5A	c.611+3A>G		Uncertain Significance
TTN	c.35794G>T	p.E11932X	Uncertain Significance

[1,2,3] Three cases were identified with two rare protein-altering variants as indicated above.

Supplementary Table 8. All rare variants and protein consequences identified in the Maastricht cohort of myocarditis patients (n=106).

Gene	Variant (HSGV)	Variant (protein)	ACMG class
BAG3	c.910C>T	Gln304*	Likely Pathogenic
DSP	c.6393del	p.Gly2133Valfs*2	Likely Pathogenic
LMNA [1]	c.992G>A	p.Arg331Gln	Likely Pathogenic
LMNA	c.1517A>C	p.His506Pro	Likely Pathogenic
RBM20 [2]	c.1900C>T	p.Arg634Trp	Likely Pathogenic
RBM20	c.1764T>G	p.Ile588Met	Likely Pathogenic
TTN	c.61921C>T	p.Arg20641*	Likely Pathogenic
TTN	c.13100del	p.Lys4367Argfs*27	Likely Pathogenic
TTN	c.73332C>A	p.Cys24444*	Likely Pathogenic
TTN	c.64688del	p.Pro21563Leufs*10	Likely Pathogenic
TTN	c.87782del	p.Pro29261Glnfs*10	Likely Pathogenic
TTN	c.13100del	p.Lys4367Argfs*27	Likely Pathogenic
TTN; LMNA	c.65042del + c.647G>A	p.Asp21681Alafs*15 + p.Arg216His	Likely Pathogenic
TNNC1 [6]	c.317+1G>A	* p.Gly68Glufs*12	Likely Pathogenic
TNNT2	c.442C>T	p.Arg148Trp	Likely Pathogenic
TNNT2	c.742T>G	p.Phe248Val	Likely Pathogenic
TNNT2	c.416G>A	p.Arg139His	Likely Pathogenic
DSC2 [5]	c.2218T>C	p.Ser740Pro	Uncertain Significance
DSG2	c.445G>A	p.Val149Ile	Uncertain Significance
DSG2 [5]	c.862G>A	p.Val288Ile	Uncertain Significance
DSG2 [8]	c.3146G>T	Arg1049Ile	Uncertain Significance
DSP [6]	c.3146C>G	p.Ser1049Trp	Uncertain Significance
DSP	c.8458T>C	p.Ser2820Pro	Uncertain Significance
DSP	c.8392A>T	p.Thr2798Ser	Uncertain Significance
DSP	c.643G>A	p.Glu215Lys	Uncertain Significance
JPH2 [2]	c.88A>T	p.Thr30Ser	Uncertain Significance
JUP [7]	c.1730G>A	p.Arg577His	Uncertain Significance
JUP [8]	c.746C>T	p.Thr249Met	Uncertain Significance
MYH7 [1]	c.3690C>T	p.Asp1230Asp	Uncertain Significance
MYH7	c.2201dupA	p.Phe735Valfs*3	Uncertain Significance
MYH7 [3]	c.3286G>T	p.Asp1096Tyr	Uncertain Significance
MYH7	c.625C>A	p.Gln209Lys	Uncertain Significance
MYH7	c.1207C>T + c.3169G>A	p.Arg403Trp + p.Gly1057Ser	Uncertain Significance
RBM20	c.2201G>A	p.Arg734Gln	Uncertain Significance
RMB20 [7]	c.2147G>A	p.Arg716Gln	Uncertain Significance
SCN5A [3]	c.6016C>G	p.Pro2006Ala	Uncertain Significance
SCN5A	c.5692C>T	p.Arg1898Cys	Uncertain Significance
SCN5A	c.647C>T	p.Ser216Leu	Uncertain Significance
SCN5A [4]	c.1858C>T	p.Arg620Cys	Uncertain Significance
TTN [4]	c.83062C>T	p.Arg27688Cys	Uncertain Significance

[1-8] Eight cases identified with two rare variants; * Protein consequence of *TNNC1* splice variant predicted using RNA sequencing (r.203_317del leading to p.Gly68Glufs*12)

Supplementary Figure 11. Histopathology and immunopathology of acute lymphocytic myocarditis in a patient found to have a rare truncating variant in *DSP*. Images are taken at 100 microns and demonstrate staining for (A) haematoxylin-eosin for nuclear and cytoplasmic components, (B) CD45 for lymphocytes, (C) CD68 for macrophages, and (D) Sirius red for collagen and fibrosis.

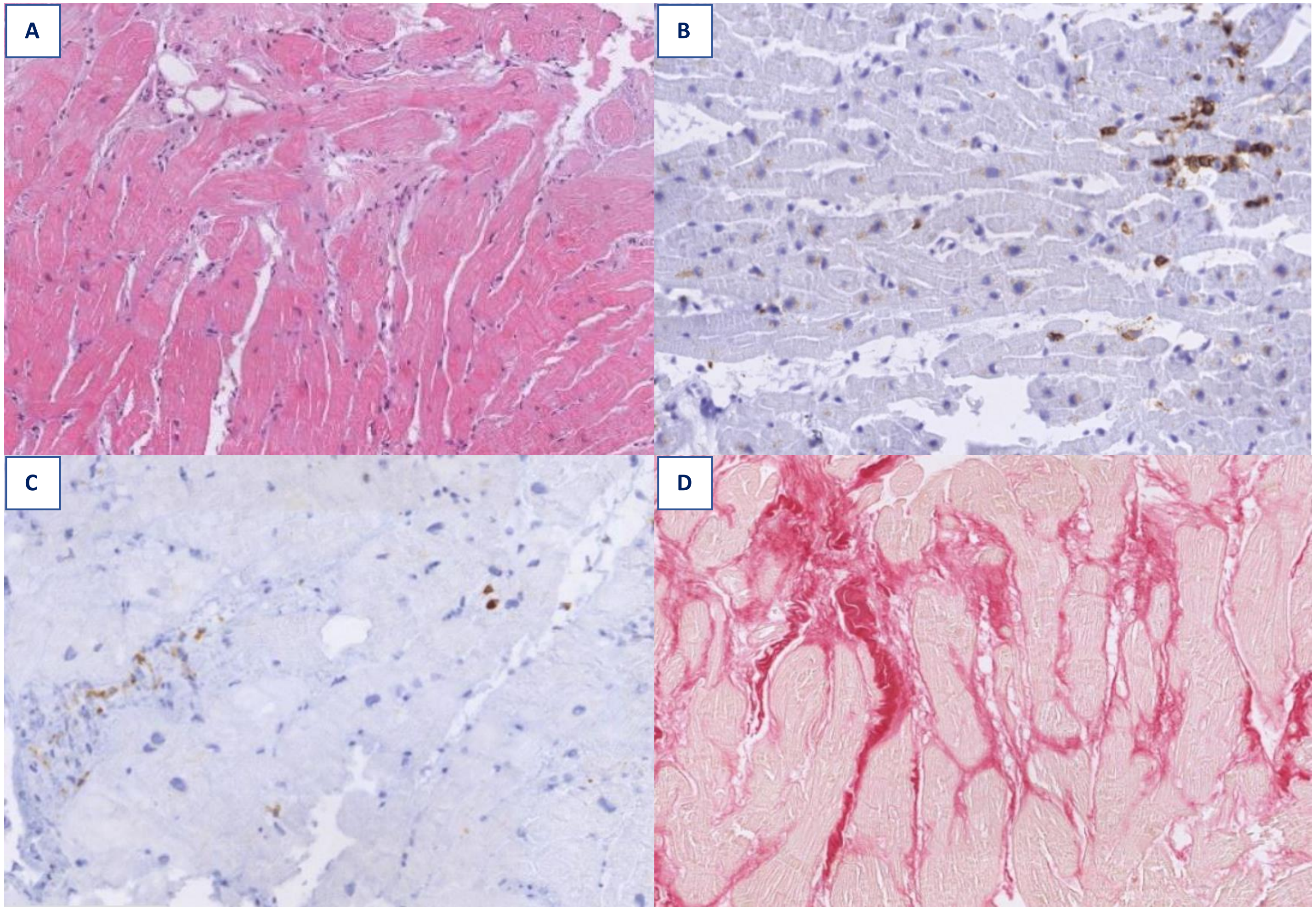

Supplementary Figure 12. All protein-altering variants in ACM and DCM genes found in the London myocarditis cohort assigned as variants of unknown significance (VUS) or likely pathogenic (LP) based on ACMG criteria applied through a semi-automated computational decision-support tool (CardioClassifier).

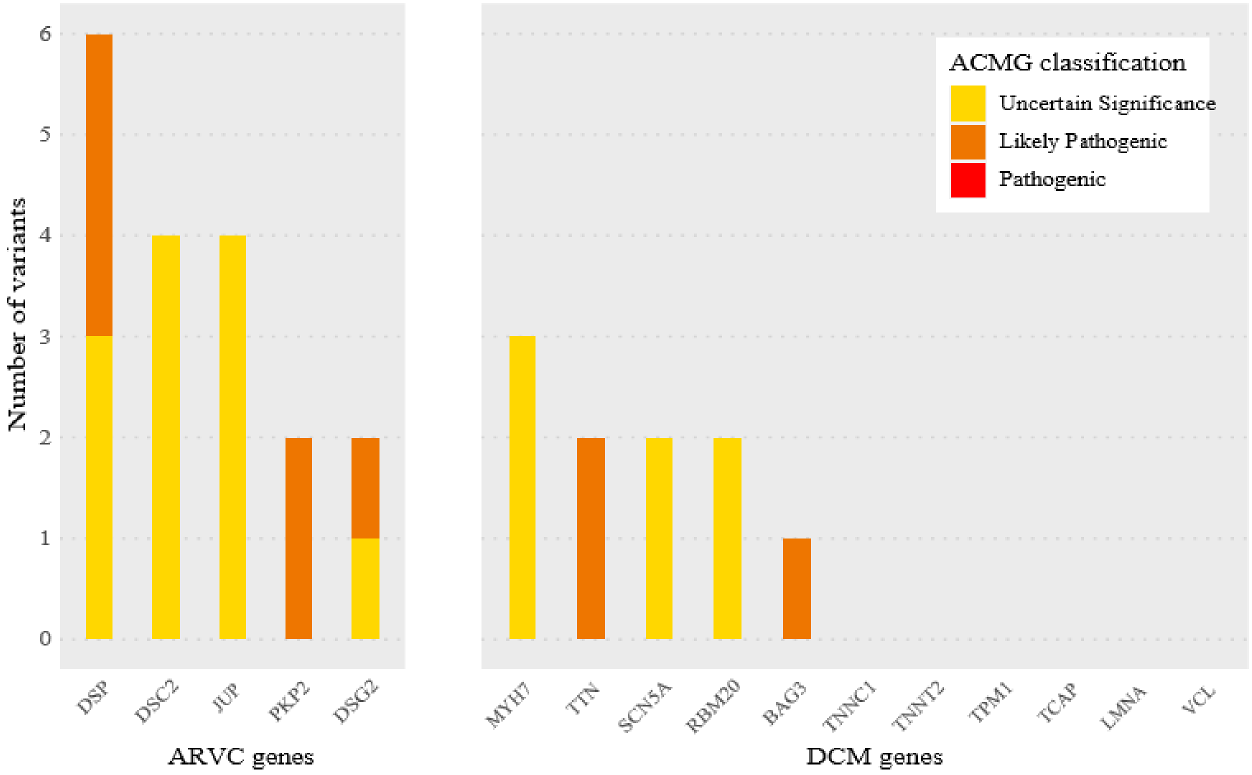

Supplementary Table 9. Clinical characteristics and outcomes of acute myocarditis cases with and without protein-altering variants in DCM- or ACM-associated genes across both cohorts (unadjusted analyses)

	**No Variant** (n=259)	**DCM variant** (n=49)	**Odds Ratio**	**P value**	**ACM variant** (n=28)	**Odds ratio**	**P value**
Baseline:							
Age	38 (26-51)	52 (37-59)		0.001	39 (27-56)		0.596
Men	81%	75%	0.72 (0.33-1.56)	0.400	64%	0.42 (0.18-1.01)	0.068
NYHA III-IV	14%	54%	3.87 (1.84-8.14)	0.001	4%	0.30 (0.04-2.29)	0.331
LVEF % (median/IQR)	60 (46-66)	39 (26-54)		<0.0001	58 (52-63)		0.739
Follow-up:							
Device implant	8%	54%	6.38 (2.9-14.04)	<0.0001	14%	1.62 (0.45-5.85)	0.441
Life-threatening arrhythmia	6%	14%	2.27 (0.78-6.58)	0.167	14%	2.16 (0.59-8.01)	0.210
HF hospitalisation	8%	25%	2.96 (1.21-7.24)	0.036	4%	0.49 (0.06-3.84)	0.706
Death	4%	18%	4.16 (1.44-11.96)	0.013	4%	0.98 (0.12-7.93)	1.000

Additional Phenotypic Data

Supplementary Figure 13. DSP frameshift c.6393del (London)

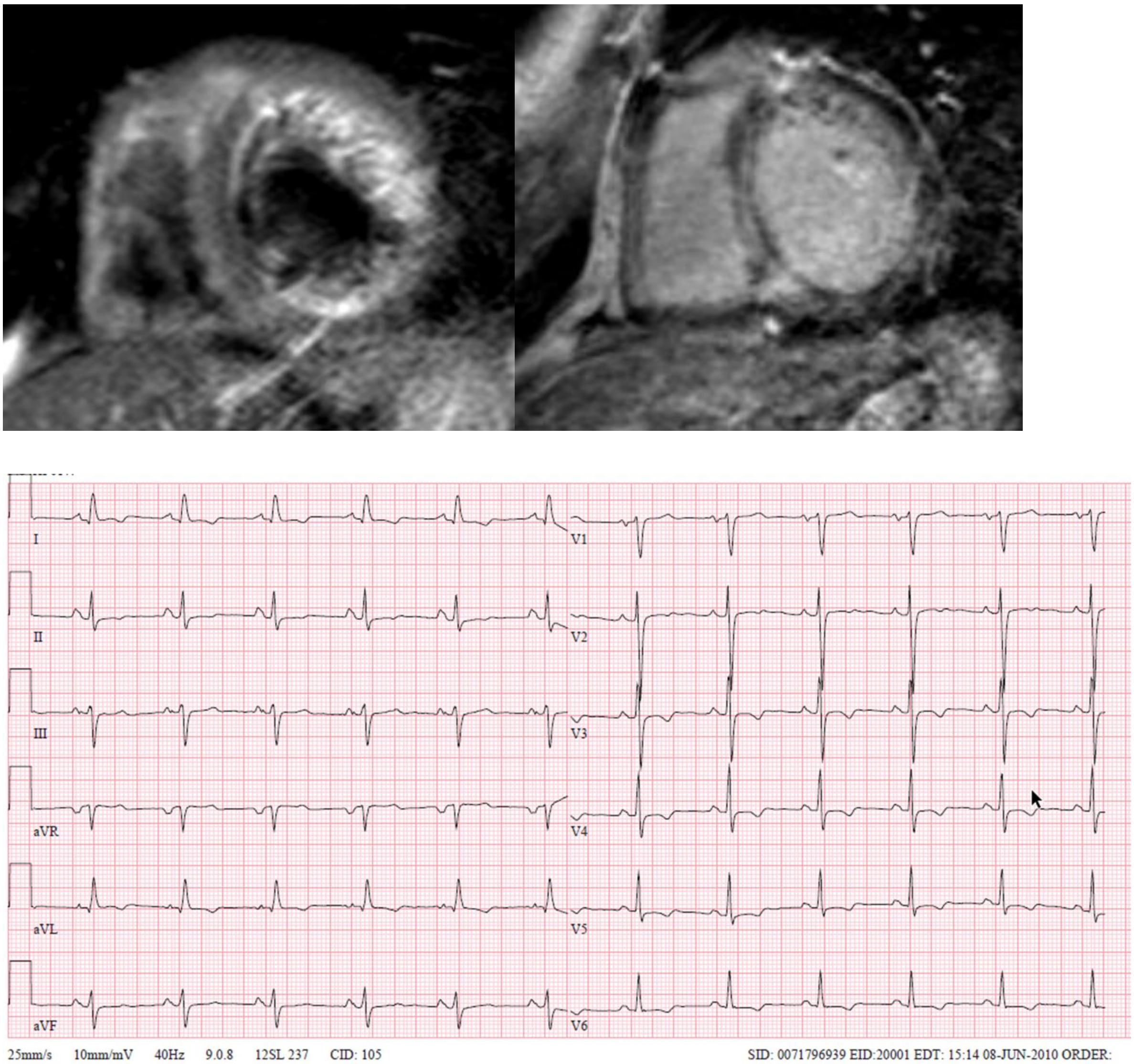

Supplementary Figure 14. DSP Frameshift c.4423del (London)

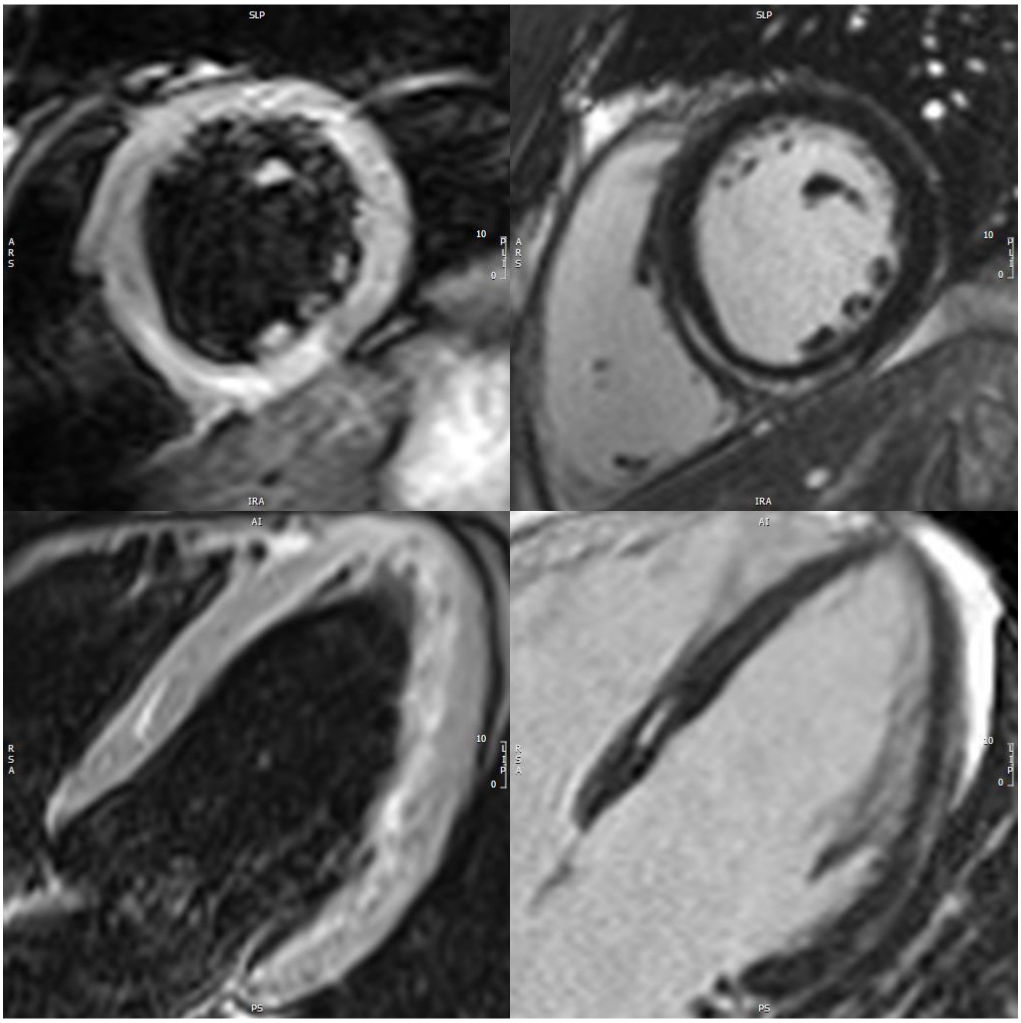

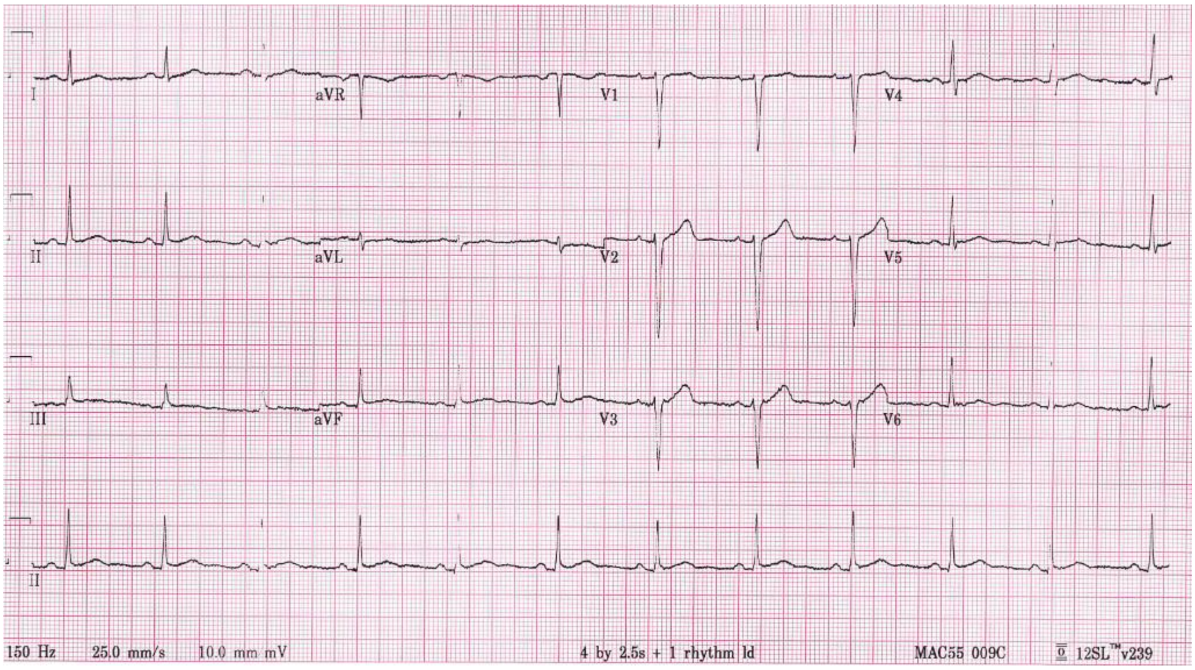

Supplementary Figure 15. DSP Frameshift c.4307_4308del (London)

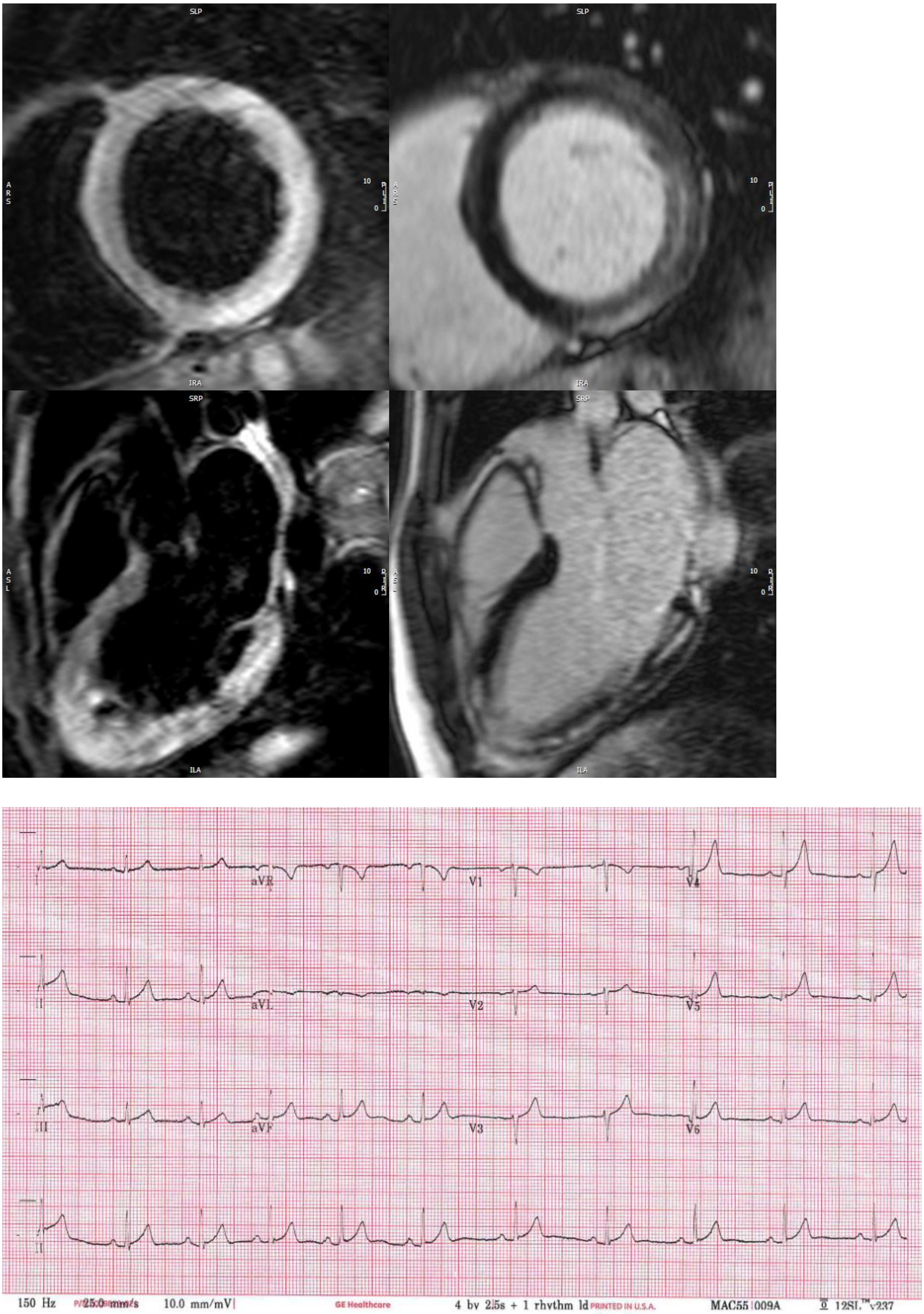

Supplementary Figure 16. DSP Nonsense c.5056C>T (Maastricht)

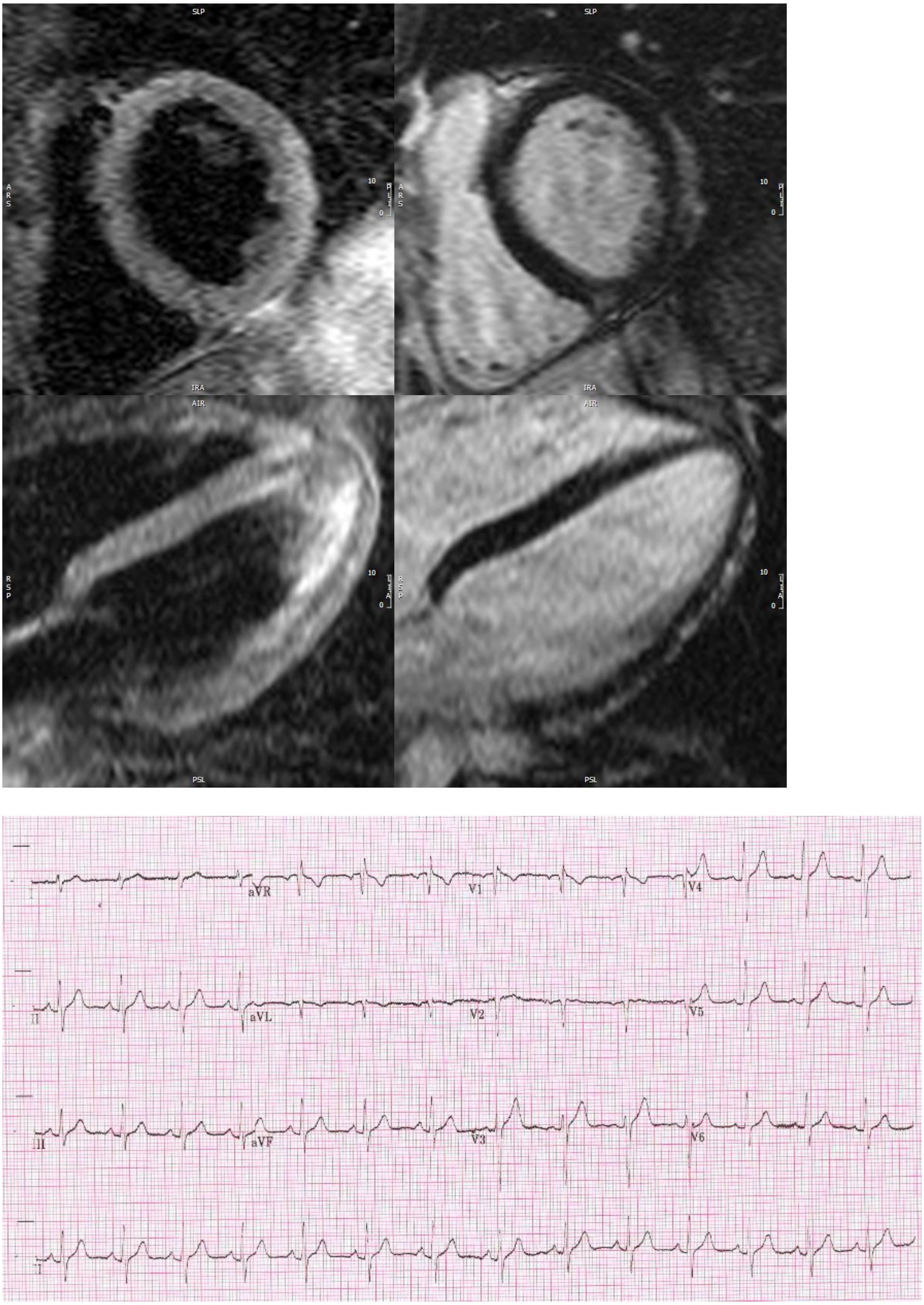

Supplementary Figure 17. Boxplot to show serial change in LVEDVi in our prospective London myocarditis cohort (n=114) across the three study time points. No significant difference was observed in any LV parameter.

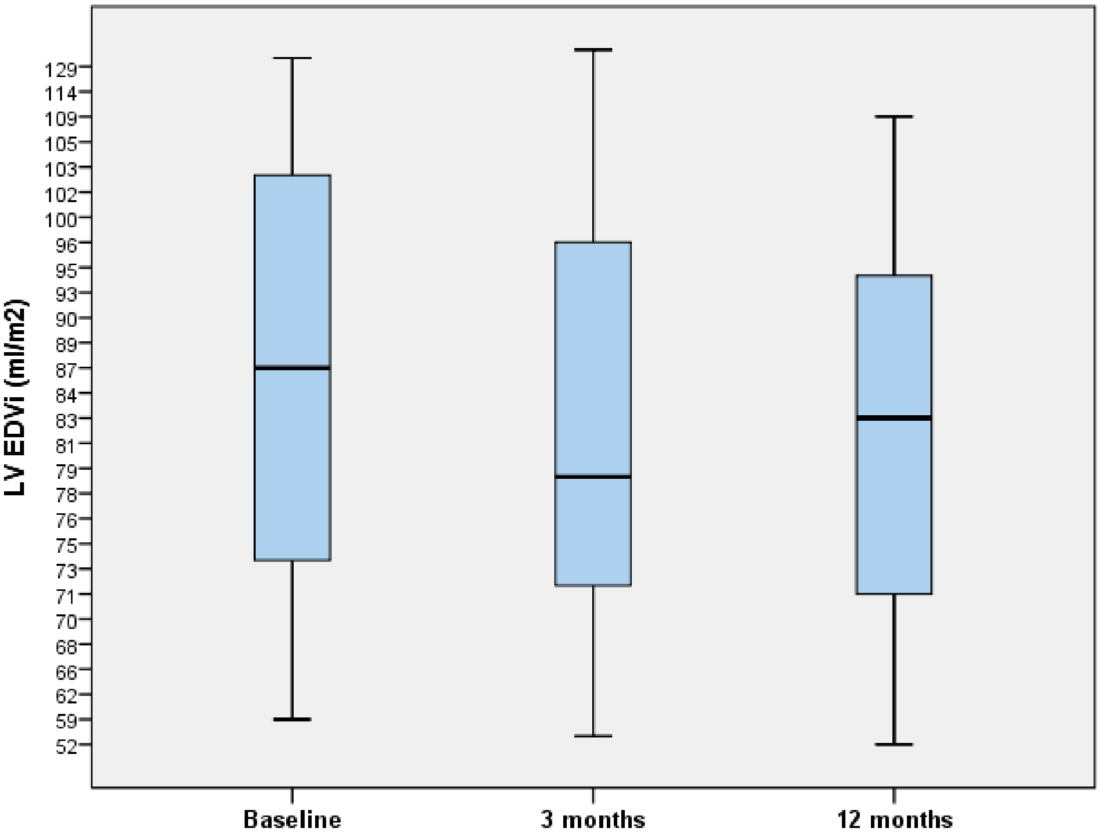

Supplement: Supplementary file 1 [file cir-146-1123-s001.pdf]
